# Small Molecule Inhibitors of Lymphocyte Perforin as Focused Immunosuppressants for Infection and Autoimmunity

**DOI:** 10.1021/acs.jmedchem.2c01338

**Published:** 2022-10-20

**Authors:** Julie A. Spicer, Kristiina M. Huttunen, Jiney Jose, Ivo Dimitrov, Hedieh Akhlaghi, Vivien R. Sutton, Ilia Voskoboinik, Joseph Trapani

**Affiliations:** † Auckland Cancer Society Research Centre, Faculty of Medical and Health Sciences, The University of Auckland, Private Bag 92019, Auckland 1142, New Zealand; ‡ Maurice Wilkins Centre for Molecular Biodiscovery, A New Zealand Centre for Research Excellence, Auckland 1142, New Zealand; § School of Pharmacy, Faculty of Health Sciences, University of Eastern Finland, P.O. Box 1627, FI-70211 Kuopio, Finland; ∥ Cancer Immunology Program, Peter MacCallum Cancer Centre, 305 Grattan Street, Melbourne, Victoria 3000, Australia; ⊥ Sir Peter MacCallum Department of Oncology, The University of Melbourne, Parkville, Victoria 3052, Australia

## Abstract

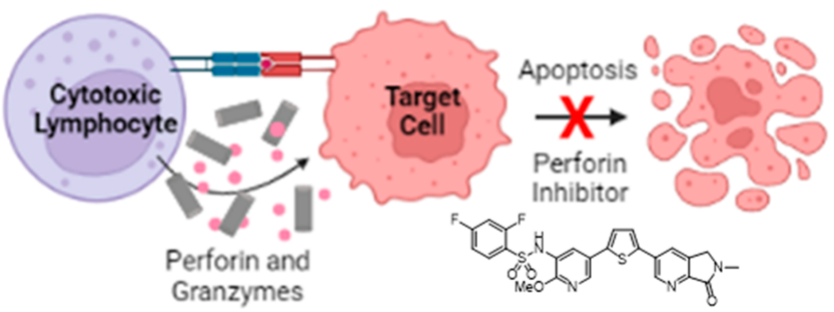

New drugs that precisely target the immune mechanisms critical for cytotoxic T lymphocyte (CTL) and natural killer (NK) cell driven pathologies are desperately needed. In this perspective, we explore the cytolytic protein perforin as a target for therapeutic intervention. Perforin plays an indispensable role in CTL/NK killing and controls a range of immune pathologies, while being encoded by a single copy gene with no redundancy of function. An immunosuppressant targeting this protein would provide the first-ever therapy focused specifically on one of the principal cell death pathways contributing to allotransplant rejection and underpinning multiple autoimmune and postinfectious diseases. No drugs that selectively block perforin-dependent cell death are currently in clinical use, so this perspective will review published novel small molecule inhibitors, concluding with *in vivo* proof-of-concept experiments performed in mouse models of perforin-mediated immune pathologies that provide a potential pathway toward a clinically useful therapeutic agent.

## 1 Introduction

Robust T cell mediated immunity is critical for the health of all mammals, as it provides protection against intracellular pathogens (especially viruses) and some cancers.^1^ However, serious pathology also arises when the immune system becomes “overstimulated” following infection (“postinfectious immunopathology”) or attacks normal self-antigens (autoimmunity). The rejection of a transplanted solid organ or bone marrow stem cells across a histocompatibility barrier (a different “tissue type”) is another setting in which cellular immunity results in adverse clinical outcomes.^2^ These disease states are typically treated with “broad spectrum” immune-suppressant drugs such as corticosteroids whose broad range of off-target effects cause considerable morbidity and some mortality.^3^ New drugs that precisely target the immune mechanisms critical for a given pathology are desperately needed. For T cell mediated diseases, the drugs should specifically block T cell functions such as cytotoxicity but allow the rest of the immune system to operate normally.

Over recent years, a range of small molecule drugs that block perforin, the key mediator of tissue damage inflicted by “killer lymphocytes”, CD8^+^ cytotoxic T lymphocytes (CTLs) and natural killer (NK) cells, have been published in the scientific literature. Both cell types are able to kill target cells perceived to be “dangerous” by the immune system, through a contact-dependent mechanism.^1,4^ In the case of CTLs, clonotypic receptors on the surface of the cell recognize a foreign or mutant peptide antigen sampled from the interior of the cell and presented on major histocompatibility complex (MHC) proteins of the target cell surface.

**Figure 1:**
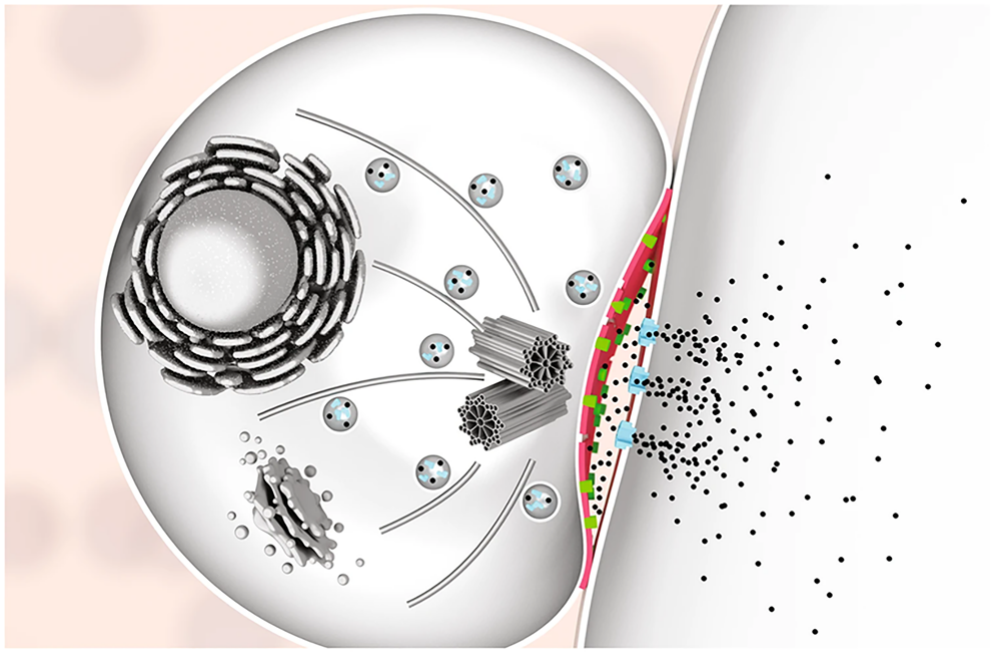
Mechanism of perforin-dependent cell killing. Formation of an immune synapse between a cytotoxic lymphocyte and a target cell results in granzymes (black dots) entering the target cells through perforin pores (light blue) in the target cell plasma membrane. Also shown is the microtubule-organizing center that enables cytotoxic granule polarization to the cell membrane where perforin and the granzymes are released into the synapse. The lymphocyte is protected from the secreted perforin by high lipid order domains (red) and exposed phosphatidylserine (green) at the immunological synapse. Reprinted with permission from ref (5). Copyright 2019 Springer Nature.

If the binding is sufficiently avid, receptor clustering and subsequent signaling^6^ result in rearrangement of the CTL actin cytoskeleton, and a stable immune synapse is formed with the target cell (Figure 1). Preformed, highly specialized cytotoxic secretory vesicles (CSVs) in the CTL cytoplasm are then recruited, migrate along the microtubular apparatus to the site of cell–cell contact, and release a cocktail of cytotoxins into the immune synapse by exocytosis.^7^ As explained below, perforin sits at the apex of a complex molecular signaling cascade that then rapidly induces the target cell to undergo programmed cell death via apoptosis.

## 2 The Structure and Function of Perforin

The toxins stored in CSVs are highly potent and bring about the death of a target cell in only a few minutes. The granule contents are diverse, but the three major constituents are perforin, a pore-forming protein whose structure and mechanism of action has been studied for many years and for which the X-ray crystal structure is solved;^5,8−16^ a family of pro-apoptotic serine proteases (“granzymes”), the most potent of which is granzyme B; and chondroitin sulfate proteoglycans, which are nontoxic but together with an acidic pH (ca. 5.0) play a key role in directing perforin and granzymes to the CSVs and in stabilizing the vesicles.^11,17^ Perforin is completely inactive prior to its export to secretory granules due to N-glycosylation of the C-terminus at Asn549 that prevents oligomerization of the perforin monomers and subsequent pore formation, thereby protecting the host cell from toxicity. Removal of this glycan within the CSVs through proteolytic cleavage activates perforin; however, it remains functionally inert due to the acidic pH of the granule. Full activation occurs when perforin is released into the neutral pH environment of the immune synapse, where the concentration of free Ca^2+^ is high (>1.0 mM), fulfilling two further requirements for perforin action on the target cell membrane.^9,11,18^

Perforin and granzyme B bring about target cell death through an elegant mechanism of molecular synergy. Perforin has no enzymic function, but upon binding Ca^2+^ in the immune synapse, perforin monomers acquire avidity for plasma membrane lipids, commencing a cascade of membrane insertion and subsequent polymerization into the mature pore form.^19^ Cryo-EM and atomic force microscopy studies showed that, following Ca^2+^ binding at perforin’s C2 domain, approximately 24 perforin monomers coalesce into ∼20 nm pores that bridge the target cell membrane, enabling granzyme diffusion into the cytosol^17^ (Figure 1). Granzyme B shares with effector caspases the unusual preference for substrate cleavage after specific acidic (aspartate) residues and, depending on the cellular context, can bring about cell death either through direct pro-caspase processing or cleavage of the BH3 adaptor protein Bid, which then activates the endogenous (mitochondrial) apoptotic pathway.^20^ While granzyme B is by far the most potent pro-apoptotic granzyme in both humans and mice,^21^ inhibition of perforin is also expected to block cell death pathways attributed to other granzymes, particularly granzyme A^22,23^ and M.^24^

As discussed in detail elsewhere,^8^ while the precise mechanism of granzyme delivery to the target cell cytosol has been the topic of conjecture over many years, the simplest mechanism in which the perforin pore facilitates granzyme diffusion into the cytosol^17^ is the most strongly supported. The diameter of the perforin pore (20 nm) is more than sufficient to admit a monomeric granzyme such as granzyme B, whose diameter is estimated from crystal studies to be 4–5 nm. In previous studies,^13^ it was shown that, in authentic immune synapses, perforin pores enable the diffusion of membrane impermeable dyes into the target cell, with the dyes spreading into the cytosol in a fashion consistent with diffusion. The main alternative hypothesis is more complex and proposes that perforin pores are internalized into endosomes in the target cell, through which the granzymes then exit into the cytosol. In the absence of perforin, recombinant granzyme B does enter cells via either clathrin-dependent or fluid phase endocytosis^25^ but remains innocuous and is eventually degraded.^26^ It has also been shown that bacterial toxins such as listeriolysin (which unlike perforin remain active at the acidic endosomal pH) are capable of delivering recombinant granzyme B to induce target cell apoptosis.^27^ More complex hypotheses propose that granzymes are internalized on far larger macromolecular complexes with chondroitin sulfate proteoglycans, a major component of cytotoxic granzymes.^28^ The interested reader is referred to other experimental approaches proposing endocytic delivery.^29,30^

Perforin plays an indispensable role in CTL/NK killing and target cell death. This is evident from the observation that the CTL/NK cells of perforin gene deleted mice or children who inherit *null* mutations affecting both perforin gene alleles have profoundly diminished cytotoxicity.^31,32^ Perforin is encoded by a single copy gene with no functional redundancy, has a unique structure, is expressed exclusively in killer lymphocytes,^8,16^ and is invariant across all human ethnicities. In most ethnicities, variations in the perforin amino acid sequence occur in fewer than 2% of individuals, and most of the 2% inherit one atypical alloform and one wild type allele. As perforin gene expression is codominant, the great majority of the residual 2% have normal (or virtually normal) CTL cytotoxic function, and perforin inhibitors will be expected to be fully effective. By far the commonest perforin polymorphism occurs mostly in Caucasians, where 8% are heterozygous for A91V, and homozygosity occurs in 1/600 individuals. This allele has significantly reduced function, but only 0.16% of Caucasians inherit two variant alleles.

These factors all suggest that perforin is a valid target for therapeutic intervention and that perforin inhibitors should cause little in the way of “off-target” effects on other tissues. The preclinical studies discussed in this perspective across several mouse models of human disease also confirm pore formation by perforin to be “druggable”. Nonetheless, perforin has until now been largely overlooked as a target for blocking CTL/NK-mediated tissue damage.

## 3 The Role of Perforin in Inflammatory Diseases

As indicated above, perforin plays a role in two broad types of immune pathology. In the first, perforin-mediated cell death mechanisms are mobilized in response to an “authentic” extrinsic immune stimulus, usually a pathogenic virus, on other occasions a parasitic organism that infects macrophages. However, the immune response is either qualitatively or quantitatively inappropriate and causes tissue damage. For example, CTL responses raised against coxsackieviruses that infect cardiac muscle cells can cause collateral damage to the myocardium with loss of muscle mass resulting in heart failure.^33−35^ In other disease settings, perforin-mediated death of macrophages infected with the protozoal parasite *Leishmania braziliensis* results in chronic skin inflammation and ulceration that persists for many months after clearance of the pathogen with antiparasitic drugs.^36^ Another example of infection-associated immunity leading to a deleterious (potentially fatal) immune-mediated outcome for the host is fulminant liver failure that occurs in a minority of human patients with acute hepatitis B infection. While most infected individuals develop jaundice, have elevated circulating liver transaminase levels but survive the infection, 5–10% raise a CTL-mediated immune response that is so rapid that it results in virtually total loss of hepatocyte mass, leading to acute liver failure.^37^ In a “proof-of-concept” study using a novel perforin inhibitor in a recognized mouse model of the disease, it has been shown that partial (50–70%) inhibition of perforin for just 1–2 days could attenuate the effector T cell response to greatly reduce liver damage and resultant mortality.^38^ There is also considerable evidence that fatal cerebral malaria is caused at least in part by breakdown of the blood–brain barrier by CTL attack. In this disease, it is hypothesized that the CTLs kill endothelial cells lining small cerebral blood vessels due to their “accidental” presentation of malaria antigens deposited on the surface by parasite infected red blood cells.^39^ Breakdown of the blood–brain barrier permits the malaria parasite to invade brain parenchyma, leading to encephalitis and death.

Tissue specific autoimmune diseases caused by inappropriate activation of CTLs against endogenous antigens comprise a second category of perforin-mediated immunopathology. By no means are all autoimmune diseases perforin-mediated; the tissue damage in many such as systemic lupus erythematosus (“lupus”) is not T cell mediated but results from inappropriate activation of B lymphocytes that secrete pathogenic antibodies that react with host antigens. In other instances, T cells cause serious immunopathology but utilize mechanisms other than perforin-mediated cell death. A classic example is CD4^+^ T cell mediated inflammation that destroys the myelin sheath encasing neuronal axons in the brain and peripheral nervous system, resulting in the diverse symptoms of multiple sclerosis.^40^ Despite this, some major illnesses *are* initiated through perforin-mediated killing of target cells that carry out an indispensable physiological function. Nonobese diabetic (NOD) mice spontaneously develop insulin-dependent diabetes as they age, due to autoimmune killing of insulin-secreting pancreatic β cells by pathogenic CTLs. NOD mice bred onto a perforin-null background still raise autoreactive CTLs but do not develop diabetes because the absence of perforin enables the β cells to survive CTL attack.^41^ In Susac syndrome, CTLs that detect an autoantigen destroy blood vessel endothelial cells in the retina and brain. The patients, frequently young women as occurs in many other autoimmune diseases, present with sudden loss of vision and varying neurological deficits (loss of balance, hearing loss) as a result of microhemorrhage and thrombosis in the eye and brain.^42^ Susac syndrome may be considered a relatively rare CD8-mediated subset of neuroinflammatory or demyelinating disorders typified by multiple sclerosis. Interestingly, while classic multiple sclerosis is almost invariably considered a CD4 T cell mediated disease, it has been noted since the 1980s that T cells infiltrating the brain of affected individuals are predominantly CD8+.^43,44^ Similar observations on T cell phenotype have recently been made on brain immune infiltrates in the early stages of Parkinson’s disease.^45^ However, a pathogenic role for perforin is yet to be proven in either of these diseases.

## 4 Small Molecule Inhibitors of Perforin

Over the past half-century, many classes of immunosuppressive drugs have been developed for the treatment of immune-mediated tissue injury; however, almost all possess a broad spectrum of activity and impact many off-targets, resulting in variable efficacy and side effects. These include corticosteroids (prednisolone, betamethasone), antiproliferative agents (methotrexate, cyclophosphamide), and suppressors of cytokine release (cyclosporins). By contrast, the ability to selectively target perforin and the granule exocytosis pathway with a small molecule could provide an immunosuppressive agent with many applications. While no drugs that selectively block perforin-dependent cell death are currently in clinical use, a range of small molecules in the published literature have been demonstrated to inhibit the perforin-dependent granule exocytosis pathway in various ways. However, most of these have poor selectivity, cause death of the CTL/NK cell, or are too toxic to be used systemically. It is this gap in the therapeutic arsenal of immunosuppressive agents that requires filling and that is addressed in the sections below.

### 4.1 Natural Product Based Inhibitors of Perforin

**Figure 2:**
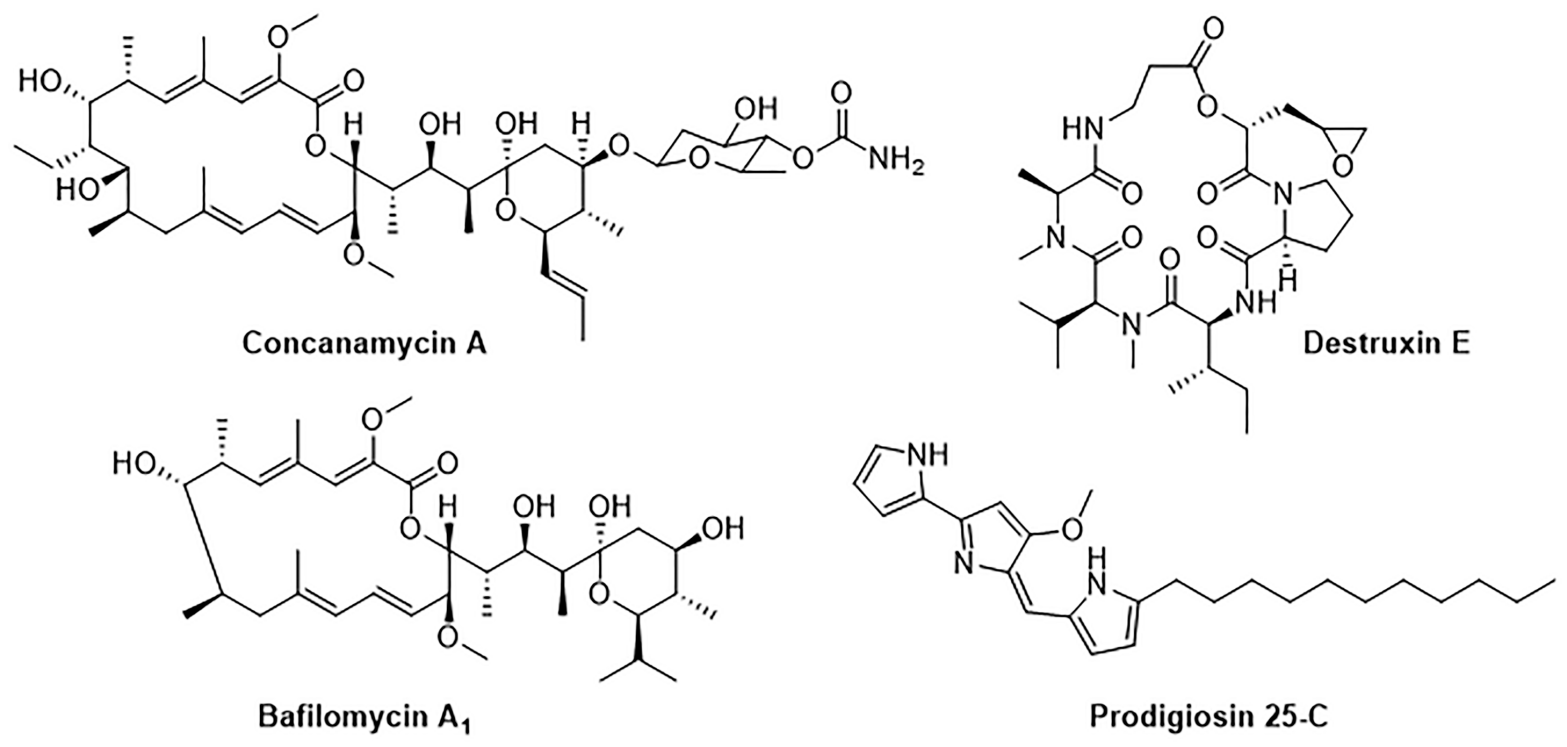
Examples of V-ATPase inhibitors.

Many complex natural products that inhibit the granule exocytosis pathway in CTLs and NK cells have been identified, but most are far too toxic or affect immune pathways other than perforin-mediated cytotoxicity to be useful clinically. These include cytochalasin D (blocks actin polymerization and prevents transferal of the cytotoxic granules to the plasma membrane); antimycin A and oligomycin A (inhibitors of cell respiration); FD-891, gliotoxin, and chebulagic acid (prevent formation of the immune synapse); and calphostin C, herbimycin A, costunolide, FK506, staurosporine, and enzastaurin (protein kinase inhibitors that block early signal transduction through the T cell receptor pathway).^46−48^ The only agents that have been shown to directly affect the concentration of perforin within the cytotoxic granules are those that act by inhibition of the vacuolar-type (H^+^) adenosinetriphosphatases (V-ATPases) that regulate lysosomal (and, therefore, CSV) pH, but these reagents adversely affect the function of many cell types other than CTLs and NK cells. Cytotoxic granules are specialized organelles that require a pH ∼5.5 within the lumen, and V-ATPases are proton pumps responsible for generating and maintaining this acidic environment.^49,50^ V-ATPase inhibitors can increase lumen pH, resulting in a significant reduction of perforin content together with morphological changes in the lytic granules.^47^ These compounds have been used extensively to probe the cytotoxic pathways employed by CTLs and NK cells *in vitro*.^47,51−55^ Examples include concanamycin A, bafilomycin A_1_, and prodigiosin 25-C, all isolated from various species of *Streptomyces* bacteria, and destruxin E, a mycotoxin derived from *Metarhizium anisopliae* (Figure 2). Concanamycin A and destruxin E are 18-membered macrolide antibiotics, and bafilomycin A_1_ is a 16-membered macrolide antibiotic, that have been used as tools to study the physiological role of V-ATPases for over 30 years.^51,56^ Prodigiosin 25-C is a fungal metabolite comprised of three conjugated pyrroles and an 11-carbon hydrophobic chain that also inhibits the acidification of lytic granules.^57^

Concanamycin A is the most well-studied V-ATPase inhibitor and has been demonstrated to block the cytotoxic activity of CTLs through an increased pH, resulting in a reduction of granular perforin content.^52,58^ Because concanamycin A selectively inhibits the perforin/granzyme mechanism of cell death and not Fas ligand based killing, it has also been used to investigate the relative contributions of these two pathways.^53,55^ It is known that perforin associates with chondroitin sulfate A at acidic pH,^59^ and it has been proposed that the increase in pH resulting from inhibition of V-ATPase by concanamycin A causes dissociation of this complex, exposing “free” perforin to serine proteases that cause hydrolysis within the cell prior to delivery.^52^ In contrast to concanamycin A (and bafilomycin A_1_), neither prodigiosin 25-C or destruxin E significantly reduced the concentration of cellular perforin; however, these agents did still block perforin activity.^54^ This observation suggested that while these inhibitors increased the intragranular pH to a point where perforin was inactivated, it was not high enough to support proteolytic degradation as in the case of concanamycin A.^54^

V-ATPase inhibitors have thus been used to dissect the various mechanisms of cell death employed by CTLs and NK cells. However, there have been no reports of natural product derived compounds that act by selectively inhibiting perforin’s action. For this reason, several classes of synthetic small molecules that block the lytic activity of perforin have been published and are discussed in section 4.2.

### 4.2 Synthetic Inhibitors of Perforin

Despite its clear importance in a normal or dysfunctional immune response as discussed above, the mechanism of perforin action at the molecular and cellular levels remained poorly understood until recently. The need for cell lines capable of synthesizing and storing inherently toxic proteins such as perforin and granzymes was first raised by Henkart and colleagues. They showed that rat basophilic leukemia (RBL) cells, which do not normally express perforin, acquired cytotoxic function if they expressed wild-type mouse perforin.^60^ This approach has subsequently been modified to define the molecular and cellular bases of the loss of function of various inherited human perforin mutations^22,61^ and to characterize the various functional domains of perforin.^15,18^

As only small amounts of perforin could be purified from RBL cells, a method for expressing mouse perforin at much higher levels by using baculovirus-infected Sf21 insect cells was devised.^62^ This methodology enabled the purification of milligram quantities of active perforin suitable for screening a chemical library of small druglike compounds to identify exemplars that could inhibit the lysis of simple cells such as sheep red blood cells (SRBCs) (Table 1). Inhibitory activity was determined at a single point concentration of 20 μM by measuring the turbidity of the reaction mixture, as detected by absorbance measurements at 650 nm. Perforin-induced lysis produced a change of turbidity that was blocked in the presence of an inhibitory compound. This primary assay resulted in the identification of 612 compounds with inhibitory activity of ≥60% at 20 μM. These compounds were then subjected to a five-point dose–response study (100, 20, 4, 0.8, and 0.16 μM) to calculate an IC_50_, resulting in 132 examples with IC_50_ values of <20 μM. Of this subset, 48 still showed robust inhibition of SRBC lysis by perforin in the presence of 0.1% bovine serum albumin (BSA). These compounds were then tested to determine if they inhibited the lysis of nucleated (Jurkat T lymphoma) cells. This was done by measuring the release of ^51^Cr from labeled Jurkat cells at four different concentrations (80, 20, 5, 1 μM). Nine compounds were found to inhibit perforin activity by ≥60% at 1 μM.^62^

**Table 1:** High-Throughput Screen (HTS) to Identify Inhibitors of Recombinant Perforin

no. of cmpds	inhibition of SRBC lysis ≥60% at 20 μM^a^	inhibition of SRBC lysis IC_50_ ≤ 20 μM^b^	inhibition of Jurkat cell lysis ≥60% at 80 μM^c^	inhibition of Jurkat cell lysis ≥60% at 1 μM^d^
101024	612	132	30	9

a Inhibition of recombinant perforin-mediated lysis of SRBC by compounds at a final concentration of 20 μM as determined by measuring cell turbidity detected by absorbance at a wavelength of 650 nM.

b An IC_50_ was calculated from a five-point dose–response curve from compound concentrations of 100, 20, 4, 0.8, and 0.16 μM.

c Inhibition of recombinant perforin-mediated lysis of nucleated (Jurkat T lymphoma) cells by compounds at a final concentration of 80 μM as determined by a ^51^Cr release assay in the presence of 0.1% bovine serum albumin.

d Inhibition of recombinant perforin-mediated lysis of nucleated (Jurkat T lymphoma) cells by compounds at a final concentration of 1 μM as determined by a ^51^Cr release assay in the presence of 0.1% bovine serum albumin.

Further studies were then conducted to test for compound specificity. To determine whether inhibitors selectively inhibited perforin, or pore-forming proteins in general, the top 105 inhibitors were tested at 20 μM for their ability to block pneumococcal toxin pneumolysin (PLO)-induced lysis of SRBC. No compound had a significant inhibitory effect, showing that they specifically inhibited perforin and not the mechanistically related PLO. This distinction was important, as structural studies have shown that PLO and related bacterial pore-forming toxins such as streptomycin and listeriolysin share a similar MACPf fold (and, yet, minimal amino acid identity) with perforin.^63^ The terminal membrane attack complex (MAC) complement components C5b, C6, and C7–9 of mammals also all share a MACPf fold with perforin. However, none of the complement components or the distantly related bacteriocidal macrophage protein perforin-2^64^ have a C2 domain. Other more distant human pore-forming proteins such as gasdermin D^65^ and antimicrobial toxin granulysin^66^ have no structural similarity to perforin and are not blocked by perforin inhibitors.

Since the original high-throughput screen was performed with mouse perforin, 17 compounds were also tested for their ability to inhibit human perforin-mediated lysis of SRBC in case a species-specific effect was evident. This was not the case, with each compound able to inhibit human perforin with potency approximately equal to or greater than that for the murine protein.

While perforin-dependent granule exocytosis is crucial to CTL and NK cell cytotoxicity, there are alternative death-receptor-mediated pathways that are mediated through Fas ligand or TRAIL (tumor necrosis factor-related apoptosis-inducing ligand). It was confirmed that eight selected inhibitors were perforin-specific and did not block cell death through these pathways. Finally, three compounds were tested for their ability to inhibit the activity of intact primary human NK cells. The NK cells were isolated from buffy coats of healthy blood donors and employed in an assay to lyse ^51^Cr-labeled K562 (leukemia) target cells. All three compounds showed the ability to block ^51^Cr release from the target cells, demonstrating their capacity to inhibit perforin delivered by intact human NK cells.

**Figure 3:**
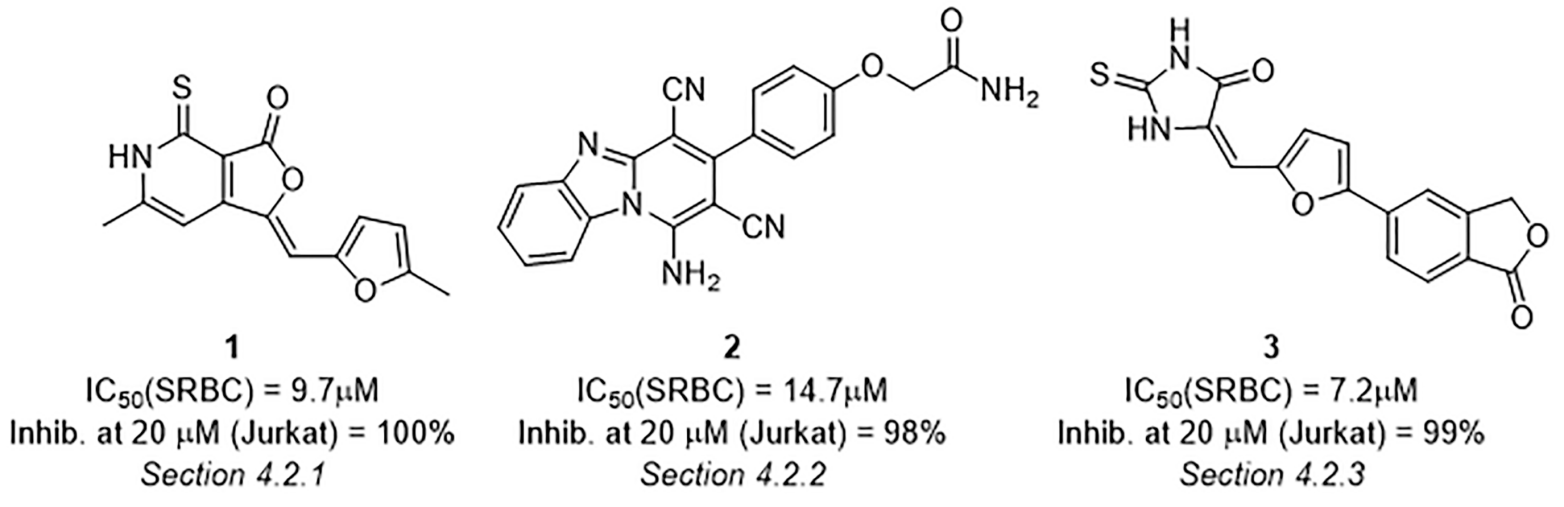
Top three validated hits from the high-throughput screen.

Ultimately the nine compounds that inhibited perforin activity by ≥60% at 1 μM in the Jurkat assay were selected for further study. To verify compound structure and activity, all were purchased from commercial vendors, tested for activity against perforin-induced lysis of ^51^Cr-labeled Jurkat cells, repurified, retested, and finally resynthesized and tested. This process resulting in three validated hits (**1**, **2**, **3**, Figure 3) that were explored further and are described in sections 4.2.1, 4.2.2, and 4.2.3.

#### 4.2.1 Dihydrofuro[3,4-*c*]pyridines

**Figure 4:**
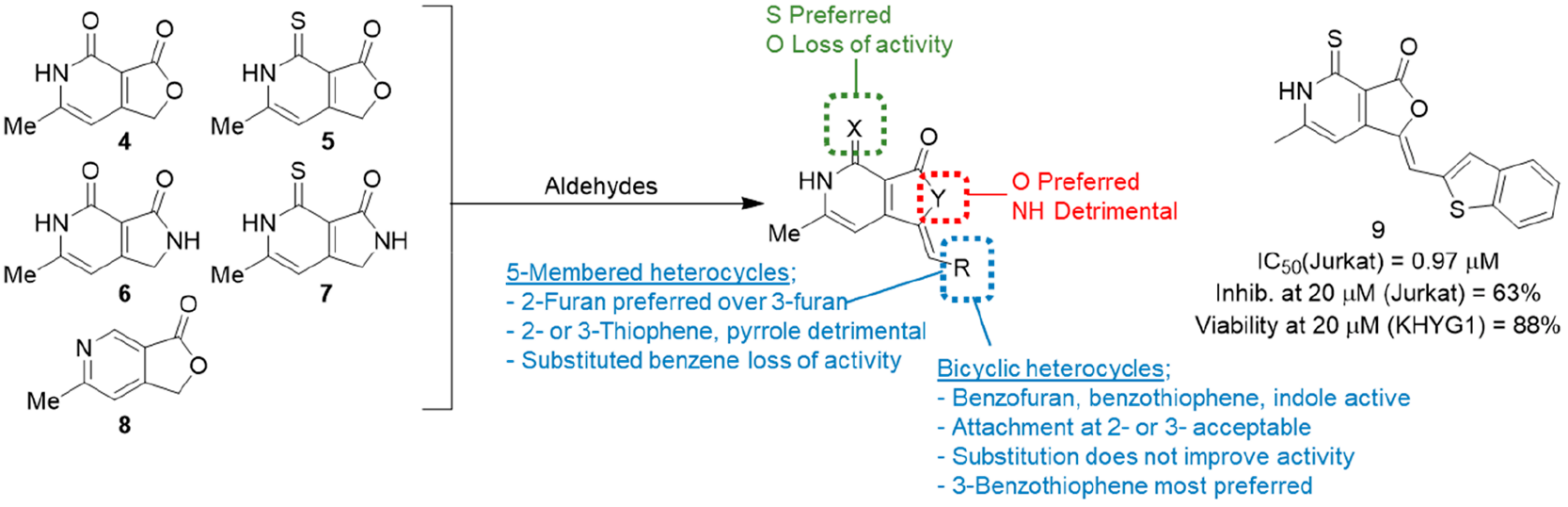
Summary of dihydrofuro[3,4-*c*]pyridine structure–activity relationship (SAR) and preferred analogue **9**.

Based on the validated HTS hit **1**, a series of dihydrofuro[3,4-*c*]pyridines were the first class of small molecule inhibitors of perforin-induced cell lysis to be disclosed.^67^ Five key bicyclic (**4**–**8**, Figure 4) cores were prepared and reacted with a variety of aldehydes in an aldolization–crotonization reaction, using piperidine catalysis under multiparallel conditions with mass spectral monitoring. The stereoselectivity of the reaction was reported to favor the *Z*-isomer based on rotational nuclear Overhauser effect (ROESY) NMR experiments supported by HPLC analysis of a representative compound. With the exception of the furopyridinone template **8**, all reactions proceeded in moderate to excellent yields, affording 53 novel compounds. All compounds were then tested in a primary assay using recombinant perforin to lyse ^51^Cr-labeled Jurkat T lymphoma cells. The lead compound **1** showed significant potency in this assay with IC_50_ = 2.7 μM. Initial analogues involved maintaining the dihydrofuro[3,4-*c*]pyridine core while varying the pendant ethylidene-linked ring (blue, Figure 4). This was in part due to concerns about the mutagenic potential of two furan-containing analogues that tested positive in the Ames *Salmonella typhimurium* TA100 strain. Monocyclic furan replacements included thiophene, pyrrole, and benzene substituted with a variety of electron-withdrawing and electron-donating groups. None of these compounds was an improvement on **1**, with the closest being a 5-phenyl-substituted thiophene (IC_50_ = 3.4 μM) and the remainder being largely inactive. Bicyclic furan replacements proved more successful, with benzofuran, benzothiophene, and indole all showing activity. Unlike the monocyclic examples, there was also more flexibility observed around the point of attachment; for example, the 2- and 3-linked benzothiophenes, benzofurans, and indoles all showed activity. Methoxy substitution on the 2-benzofuran was beneficial (IC_50_ = 8.2 μM for the unsubstituted analogue compared to 6.7, 2.4, 7.2, and 3.0 μM, respectively, for the 4-, 5-, 6-, and 7-OMe analogues); however, for the most part this did not extend to the benzothiophenes or indoles. The dihydrofuro[3,4-*c*]pyridine core was also modified (green and red, Figure 4), and it was found that replacement of the sulfur with oxygen generally gave less potent compounds (green). Likewise, replacement of the lactone, a potential metabolic lability, with a lactam resulted in loss of activity (red).

Selected analogues were then chosen for further analysis. Lead compound **1** was found to not inhibit the pneumococcal toxin PLO or the FasL/TRAIL-mediated cell death pathways, providing supporting evidence for the inhibitors being on-target for perforin. Compound **1** was also shown to inhibit perforin activity in primary human NK cells isolated from healthy blood donors; by using ^51^Cr-labeled K562 target cells, the release of ^51^Cr resulting from cell lysis was blocked by >70%.

Five novel compounds with IC_50_ values of <5 μM were then selected for further analysis. This included testing for their activity in an assay that employed an intact NK cell line to deliver perforin under more physiologically relevant conditions than the primary screen using isolated recombinant perforin. Human KHYG1 NK cells and medium were coincubated with inhibitor at a final concentration of 20 μM. ^51^Cr-labeled K562 leukemia target cells were added, and after 4 h cell lysis was measured by ^51^Cr release. All five compounds inhibited NK cell killing of the K562 targets, ranging from 50 to 100%. It was also necessary to establish that the apparent blocking of NK cell activity was not due to nonspecific toxicity toward the effector NK cell, so the viability of the NK cells in the presence of inhibitor was measured 24 h later. While three of the five compounds were toxic to the NK cells, the remaining two and the lead **1** proved that it was possible to inhibit NK cell activity yet largely retain NK cell viability. This led to the identification of **1** and **9** as the most potent compounds overall, as they possessed activity against both recombinant perforin (IC_50_ = 2.7 and 0.97 μM, respectively) and perforin delivered by NK cells (50 and 63% inhibition at 20 μM) while largely retaining NK cell viability (72 and 88%).

#### 4.2.2 Amino-2,3-dicyanopyrido[1,2-*a*]benzimidazoles

**Figure 5:**
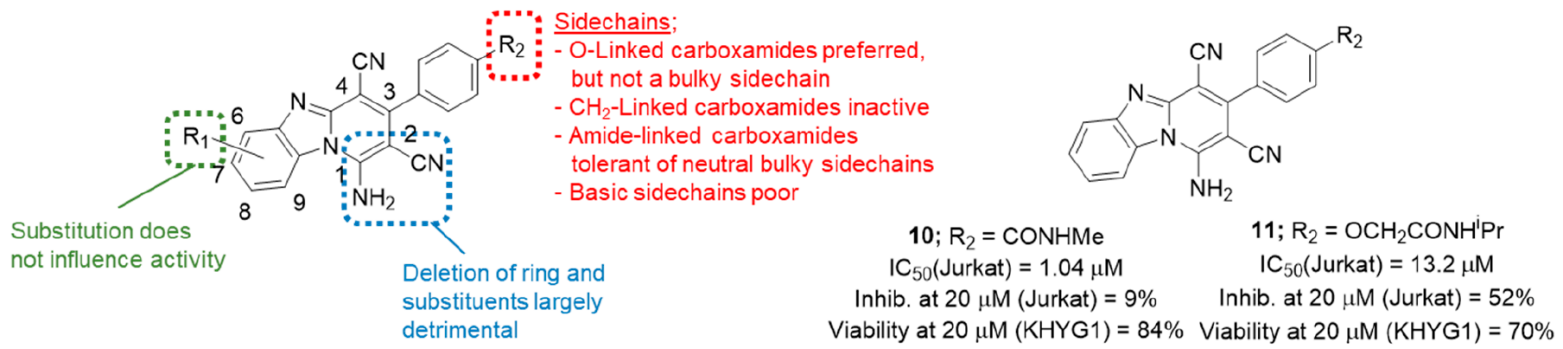
Summary of amino-2,3-dicyanopyrido[1,2-*a*]benzimidazole SAR and preferred analogues **10** and **11**.

While the dihydrofuro[3,4-*c*]pyridines as exemplified by **9** were promising compounds, their activity was later shown to be adversely affected by increasing concentrations of serum which limited their utility as potential drug leads. This led to the pursuit of an alternative series: the amino-2,3-dicyanopyrido[1,2-*a*]benzimidazoles based on hit compound **2**.^68^ Targets in this class were synthesized by using minor modifications of literature methods^69−71^ with variations focusing mainly on the aryl-linked side chain (red, Figure 5).

Structure–activity relationships were developed by testing all compounds in the previously described Jurkat assay (section 4.2) using recombinant mouse perforin. The IC_50_ for the original HTS lead compound **2** was determined as a reference point and was found to be 5.19 μM. Initial analogues focused on small changes to the oxygen-linked side chain at the 4′-position of the 3-aryl group (red, Figure 5). Within this set the SAR was found to be tight, with little variation tolerated outside the primary amide of **2**, apart from the CONHMe and CONMe_2_ analogues (IC_50_ = 4.56 and 6.89 μM, respectively). Bulky substituents on the amide were found to be particularly detrimental. Activity was also completely lost when the side chain was linked via a methylene instead of oxygen. A simple amide substituent directly on the aryl group provided the most potent compound: the methylcarboxamide **10** (Figure 5). Extrapolation of this result into a series of amide-linked side chains revealed far more tolerance to substitution than for the corresponding ether-linked compounds, the only exception being strongly (dimethylamino, piperidine) or weakly (morpholine) basic amino groups. The activity of compounds containing neutral side chains with hydroxyalkyl substituents (five examples) were all tightly clustered between IC_50_ = 3.16 μM and IC_50_ = 5.55 μM. Substitution with methyl, chloro, or methoxy on the tricyclic ring system (positions 6–8) conferred no particular advantage (green, Figure 5), while reduction of the tricyclic to a bicyclic ring system gave either equivalent (two examples, IC_50_ = 6.72 and 5.14 μM) or poorer (three examples, all >20 μM) activity than **2** and were therefore not pursued further (blue, Figure 5). Twenty of the 36 compounds in the paper were then tested for activity against KHYG1 NK cells. It was discovered that the lead **2** had relatively poor activity against whole NK cells: 18% inhibition at 20 μM. This proved to be the case across the series, with none being as potent as the dihydrofuro[3,4-*c*]pyridines of section 4.2.1, and the most potent compound **11** showing 52% inhibition. Furthermore, the SARs for the inhibition of recombinant perforin and perforin produced by NK cells appeared to diverge, with poor correlation observed between the potencies of the compounds in the two assays. This was attributed to the additional barriers to compound distribution present in a cell–cell assay compared to the use of recombinant protein, especially for more insoluble compounds. In conclusion, although this series appeared to be relatively unaffected by the presence of serum, the overall lack of activity against NK cell activity resulted in it being discontinued.

#### 4.2.3 Aryl-Substituted Isobenzofuran-1(3*H*)-ones and 5-Arylidene-2-thioxoimidazolidin-4-ones

**Figure 6:**
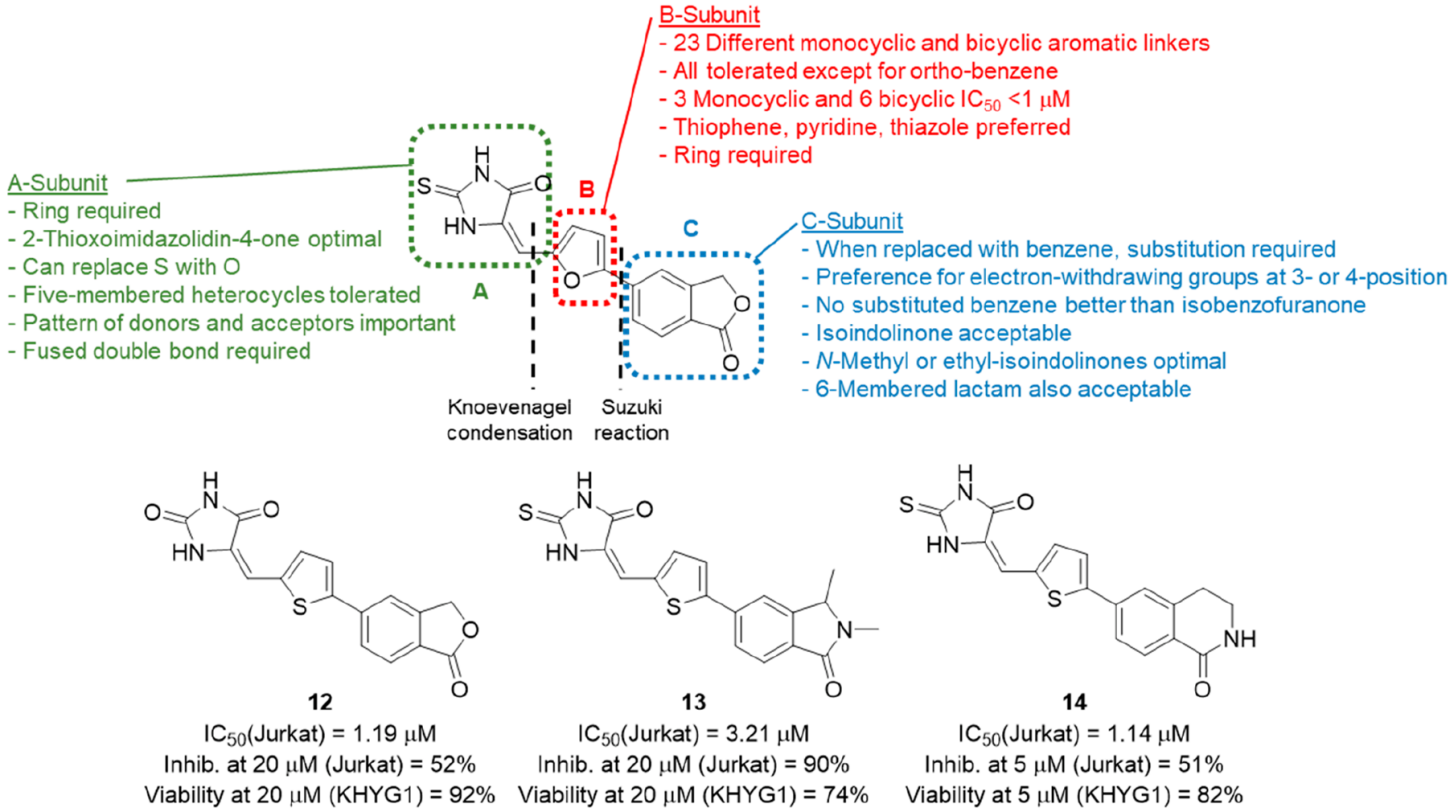
Summary of aryl-substituted isobenzofuranone SAR and preferred analogues **12**, **13**, and **14**.

With the abandonment of the amino-2,3-dicyanopyrido[1,2-*a*]benzimidazole series, the final validated hit from the original HTS, compound **3**, was investigated. Fortunately, this scaffold was amenable to disconnection into three subunits: a 2-thioxoimidazolidin-4-one (A), a furan (B), and a benzofuranone (C) (Figure 6). The compound class was therefore explored through independent variation of each individual subunit while the other two subunits remained fixed. Together this resulted in the synthesis of 111 novel compounds and the establishment of a detailed SAR for this class of perforin inhibitors.^72−74^ Generally, compound assembly was based on a key Suzuki reaction between protected aldehyde B-subunits and the C-subunit, followed by a Knoevenagel condensation between the deprotected aldehyde B–C intermediates and A-subunits containing an activated methylene group.

The initial strategy centered on retaining the 2-thioxoimidazolidin-4-one A and isobenzofuranone Csubunits, while exchanging the central furan ring for a variety of aromatic B-subunits (red, Figure 6).^73^ A total of 23 different monocyclic and bicyclic aromatic linkers were explored. In the Jurkat assay using recombinant perforin, lead compound **3** had an IC_50_ value of 6.20 μM. By simply replacing the furan B-subunit of this compound with thiophene, the IC_50_ was improved >8-fold to 0.78 μM. 2,5-Pyridyl and 2,4-thiazole were also submicromolar inhibitors of perforin activity (0.37 and 0.34 μM, respectively), as were bicyclic examples containing 2,5- and 2,6-linked benzo[*b*]thiophene, 2,5-linked benzofuran, 2,5 and 2,6-linked 1*H*-indole, and 2,6-linked quinoline (IC_50_ values between 0.36 and 0.82 μM).

On the basis of a range of factors, including inhibitory activity, synthetic accessibility, and overall molecular weight of the final compound, thiophene was selected as the preferred B-subunit. The impact of variations on the A-subunit in the context of a fixed thiophene B-subunit and isobenzofuranone C-subunit was then investigated (green, Figure 6). Acyclic analogues were found to have poor activity, suggesting the requirement for a ring as the A-subunit. Replacement of the 2-thioxoimidazolidin-4-one with a variety of closely related five-membered heterocycles (e.g., imidazolidine-2,4-dione, 2-thioxothiazolidin-4-one) and six-membered heterocycles (e.g., pyrimidine-2,4,6(1*H*,3*H*,5*H*)-trione, 2-thioxodihydropyrimidine-4,6(1*H*,5*H*)-dione) resulted in a series of active compounds (IC_50_ = 0.40–3.96 μM) with the 5-oxazole also showing moderate activity (IC_50_ = 3.18 μM). Systematic deletion of NH and carbonyl groups was then performed, resulting in loss of potency in all cases, demonstrating that the overall pattern of hydrogen bond donors and acceptors as exemplified by the 2-thioxoimidazolidin-4-one A-subunit is required for activity. This is supported by a loss of potency observed when the NH groups were substituted with a methyl group. Additionally, when the double bond fused to the C-subunit was saturated, activity was completely lost, indicating that this element is also required.

From the set of compounds that were prepared with A- and B-subunit variations,^73^ 20 out of 43 active examples (IC_50_ = 0.37–1.80 μM) were selected for further analysis by assessing their ability to inhibit perforin delivered by an intact NK cell line. The compound (20 μM final concentration) and medium were coincubated with KHYG1 NK cells, and ^51^Cr release from labeled K562 target cells was determined. Unfortunately, although all compounds were potent against isolated recombinant protein, many also proved toxic to the effector cell when whole NK cells were employed to deliver perforin. The best compound overall in this study was imidazolidine-2,4-dione, **12**, which balanced good inhibitory activity against isolated perforin (IC_50_ = 1.19 μM) with excellent suppression of KHYG1 NK cell mediated lysis in the presence of serum (64%) without toxicity toward the effector NK cells (>90% survival).

A second paper investigating lead compound **3**(74) then sought to maximize inhibitory activity and optimize “druglike” properties by varying the isobenzofuranone C-subunit in the presence of the preferred 2-thioxoimidazolidin-4-one/thiophene (AB-subunit). This gave a series of 5-arylidene-2-thioxoimidazolidin-4-ones (blue, Figure 6). Because the 2-thioxoimidazolidin-4-one A-subunit is closely related to the well-established pan-assay interference (PAIN) compound rhodanine, **3** was subjected to PAINS filters^75−77^ which it passed, likely due to the presence of an extra nitrogen in the core five-membered ring.

Replacement of the isobenzofuranone with a range of substituted benzenes showed that an electron-withdrawing group at the 3- and/or 4-position was preferred in testing against recombinant perforin. The most potent compounds were found to be 3- and 4-CONH_2_ (IC_50_ = 0.79 and 1.56 μM, respectively); however, all attempts to improve solubility through the introduction of side chains on these amides resulted in reduced activity. Overall, no substituted benzene was an improvement on the original isobenzofuranone, so an alternative strategy using the closely related isoindolin-1-one was employed instead. The rationale was that a lactam should be less susceptible to hydrolysis than the original lactone, in addition to providing a nitrogen for the introduction of further substituents capable of modifying potency and/or solubility. The parent isoindolin-1-one had reduced activity compared to the isobenzofuranone; with the IC_50_ value of 0.78 μM increasing to 2.55 μM; however, when the lactam nitrogen was substituted with simple alkyl groups, as exemplified by **13** (Figure 6), potency ranged between 0.51 μM (methyl) and 4.42 μM (isopropyl). Longer *N*-hydroxyalkyl side chains were also acceptable (IC_50_ = 0.53–1.26 μM), but basic aminoalkyl side chains were not tolerated (IC_50_ = 8.93 to >20 μM). Expansion of the five-membered isoindolin-1-one lactam ring to the six-membered ring of a 3,4-dihydroisoquinolin-1(2*H*)-one (e.g., **14**) was also tolerated, although in this case the preference was for the lactam nitrogen to remain unsubstituted. Finally, with the 2-thioxoimidazolidin-4-one A-subunit fixed, the best B-subunits of the previous paper^73^ were reintroduced to the now preferred isoindolin-1-one and 3,4-dihydroisoquinolin-1(2*H*)-one C-subunits. The 2,5-thiophene was confirmed as the optimal B-subunit across this series.

When a subset of 10 compounds with activity against isolated perforin was tested against whole KHYG1 NK cells, potency ranged between 42 and 90% inhibition at 20 μM concentration. The compounds were largely nontoxic. However, one exception was **14**, which was toxic at 20 μM (38% NK viability), so this was also tested at 5 μM, giving 51% inhibition at 82% viability. By taking into consideration potency (isolated protein and NK cells), lack of toxicity, and solubility (all compounds prepared as the sodium salts), two compounds were identified as possessing the best overall profile for *in vivo* pharmacokinetics (PK) studies (**13** and **14**). These inhibitors were evaluated for plasma PK in male CD-1 mice and were found to have acceptable drug exposures at 5 mg/kg, despite a relatively high volume of distribution and clearance. In maximum tolerated dose (MTD) studies using intraperitoneal dosing, both compounds were found to be well tolerated after single doses of 80 (**13**) and 60 mg/kg (**14**), while with multiple dosing of twice daily over 3 days, 60 and 40 mg/kg were also found to be acceptable.

Further studies were also conducted to investigate the mechanism of perforin inhibition. By use of recombinant mouse perforin immobilized via amide coupling on a surface plasmon resonance (SPR) CM-5 chip, **13** and **14** were shown to bind reversibly using SPR detection. Compound **14** was also employed in real-time microscopic imaging of the natural killer–target cell interaction. When target cells are exposed to NK cells and a synapse is formed, a sequence of downstream signaling events is triggered, resulting in an influx of Ca^2+^ into the killer cell that can be detected with the calcium ionophore Fluo-4. This influx is known to directly precede degranulation and the secretion of perforin into the synaptic cleft.^13^ Disruption of the target membrane by perforin can be detected through the incursion of extracellular propidium iodide (PI). Together, visualization of Ca^2+^ influx by Fluo-4 and membrane disruption using PI were deployed in an assay where the interaction of single NK and target cell pairs could be observed by microscopy in real time. In the presence of perforin inhibitor **14** (20 μM), only 60% of killer cells were able to kill the target cells (*n* = 50) compared to 100% death in the case of the DMSO control. This showed that **14** had the capacity to block perforin activity within the immunological synapse without affecting the stability of synapse formation, leading to the conclusion that the inhibitors are likely to act on perforin after its release into the immune synapse.

#### 4.2.4 Diarylthiophenes

While the aryl-substituted isobenzofuran-1(3*H*)-ones and 5-arylidene-2-thioxoimidazolidin-4-ones discussed in section 4.2.3 showed considerable potential and enabled further elucidation of perforin’s mechanism of action, there were several obstacles to overcome before progression to *in vivo* efficacy studies in murine disease models. Although preliminary PK studies had shown that these compounds were relatively well tolerated in healthy mice,^74^ their variable toxicity in NK cells raised concerns about advancing such compounds into the immunocompromised mice required for efficacy studies. To address this, bioisosteric replacements were sought for the 2-thioxoimidazolidin-4-one subunit, because while this moiety passed PAINS filters,^75−77^ all derivatives still contained a potentially reactive Michael acceptor and existed as interconverting mixtures of *E*- and *Z*-isomers. Improved potency was also sought to allow the delivery of a lower efficacious dose to enable better control of potential dose-related toxicity.

**Figure 7:**
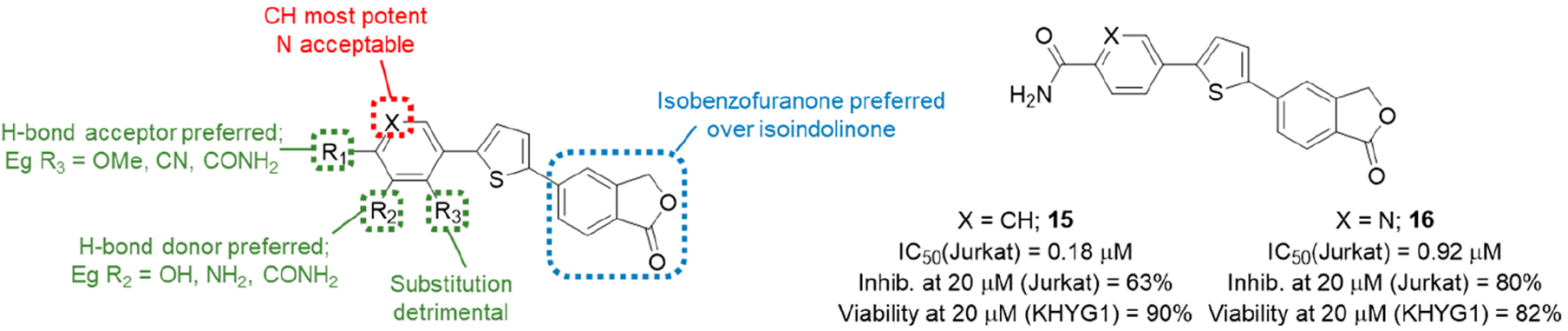
Summary of diarylthiophene SAR and preferred analogues **15** and **16**.

The proposed biososteres were comprised of a series of substituted benzene and pyridyl rings designed to occupy the same topographical space as the 2-thioxoimidazolidin-4-one pharmacophore and mimic the pattern of hydrogen bond donors (HBDs) and acceptors (HBAs) required for perforin inhibitory activity.^78^ In the first instance, methyl, methoxy, and chloro were surveyed at the 2-, 3-, and 4-positions (green; R_1_, R_2_, R_3_, Figure 7). All had no detectable activity against recombinant perforin, except for 4-OMe (R_1_ = OMe) which had a modest IC_50_ value of 9.36 μM and suggested the requirement for an HBA at this position. The series was then expanded with a larger range of substituents containing HBDs and HBAs, such as NH_2_, OH, and CN. Amino at R_1_ or R_2_ showed similar activities (IC_50_ = 11.82 and 13.97 μM, respectively); however, the SAR diverged in the cases of OH and CN. Nitrile at the 4-position gave good activity (IC_50_ = 6.87 μM), but the 3-CN was inactive, supporting the requirement for an HBA at this position, whereas for a hydroxy substituent the opposite was the case with the 4-OH inactive and the 3-OH showing good activity (IC_50_ = 5.89 μM) and suggesting the need for an HBD at this position. These findings were explored further with the preparation of the 3- and 4-CONH_2_ derivatives. Both compounds showed appreciable activity (IC_50_ = 2.97 and 0.18 μM, respectively), with the 4-carboxamide **15** the most potent compound against recombinant isolated perforin identified to date. Introduction of a nitrogen to the ring to give the corresponding pyridine-4-carboxamide **16** (red; X = N, Figure 7) also gave a highly effective inhibitor of perforin activity (IC_50_ = 0.92 μM). Combination of the preferred 3-OH and 4-CONH_2_ substituents afforded another potent compound (IC_50_ = 0.67 μM), while the 4-CH_2_OH was also submicromolar (IC_50_ = 0.90 μM). Further exploration of the 4-carboxamide showed that conversion of the primary amide of **15** to CONHMe or CONMe_2_ resulted in loss of activity, while basic solubilizing side chains were poor and neutral side chains of moderate potency (IC_50_ = 2.65–3.31 μM), with none an improvement on **15**. Replacement of the isobenzofuranone with an isoindolin-2-one or *N*-methylisoindolin-2-one (blue, Figure 7) resulted in significant (IC_50_ = 6.04 μM) or total of loss activity.

The five most potent inhibitors were then investigated further, starting with stability in mouse and human plasma. Each compound was incubated in plasma at 37 °C for 24 h followed by HPLC analysis of the parent compound remaining. All five examples were significantly more stable in mouse compared to human plasma; for example, **15** showed 75% parent remaining in mouse plasma but only 46% in human plasma. It was observed that this was an issue that would need to be addressed in future work, and a parallel was drawn with camptothecin, which also contains a lactone ring and that has been shown to coexist in the closed and ring-opened forms.^79^ When tested against whole NK cells, **15** and **16** stood out as the superior examples from this series with good potency (63 and 80% inhibition of lytic activity at 20 μM concentration, respectively) with minimal toxicity toward the effector cells (90 and 82% viability). Thus, the objective of identifying a nontoxic bioisosteric replacement for the thioxoimidazolidinones was achieved, and this series of diarylthiophenes provided a bridge to the benzenesulfonamide-based *in vivo* candidates described in section 4.2.5.

#### 4.2.5 Benzenesulfonamides

**Figure 8:**
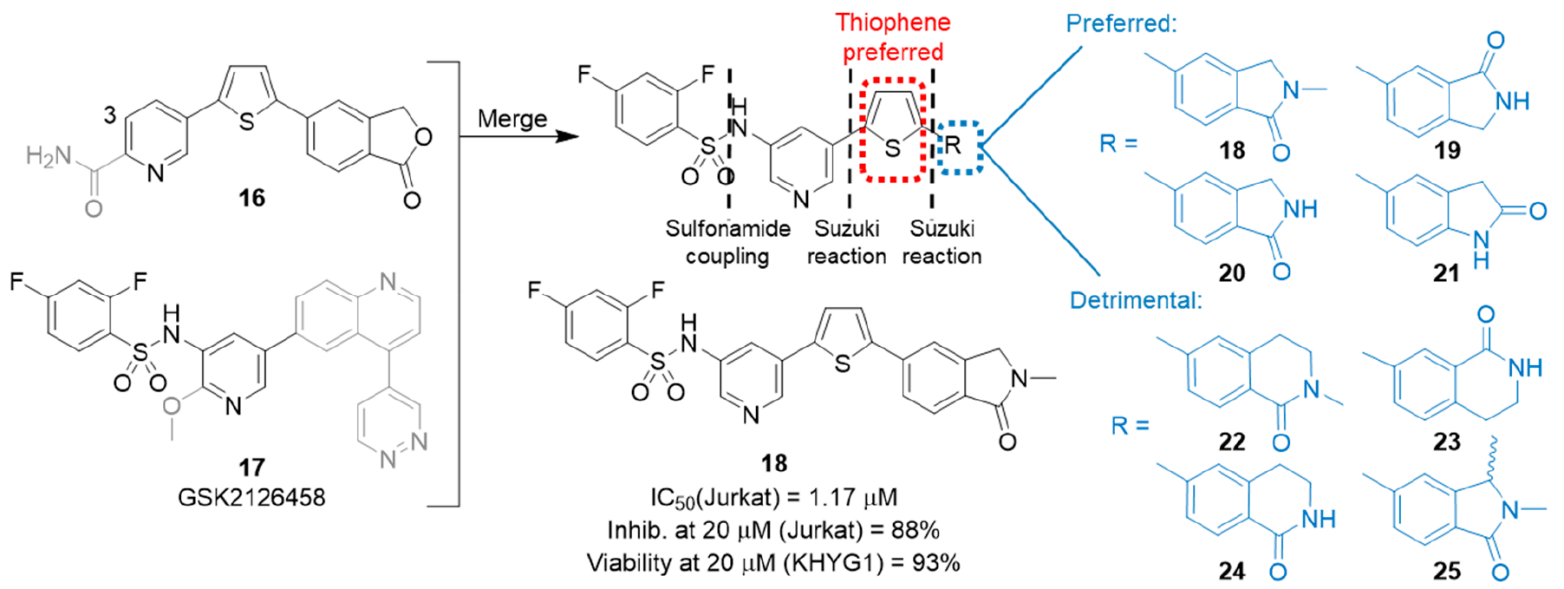
Summary of initial benzenesulfonamide strategy and preferred analogue **18**.

The diarylthiophenes of section 4.2.4 afforded several submicromolar inhibitors of recombinant perforin that also blocked NK activity with minimal toxicity; however, a major drawback of these highly aromatic structures was their extreme insolubility (0.023–16 μg/mL).^78^ This problem was addressed with a series of benzenesulfonamides that were hybrids of diarylthiophene **16** and GSK2126458 (Figure 8), an orally bioavailable inhibitor of phosphoinositide 3-kinase α (PI3Kα) and mammalian target of rapamycin (mTOR).^80,81^ GSK2126458 employed a pyridyl-linked benzenesulfonamide as a bioisosteric replacement for a thiazolidinedione in a similar manner to the thioxoimidazolidinone replacements discussed in section 4.2.4. This appeared to complement the existing diarylthiophene SAR and provided an opportunity to target more potent, soluble perforin inhibitors.

The target core was assembled through a sulfonamide coupling and two Suzuki reactions, with the exact order dependent on the individual final product. In the first instance, the 2,4-difluorobenzenesulfonamide was connected to the pyridine of **16** at the 3-position, and through a 2,5-thiophene link, a series of 14 bicyclic templates (eight highlighted in Figure 8) were introduced. The set of bicycles focused on isoindolinone-based analogues rather than the original isobenzofuranones to avoid the previously discussed tendency of the lactone to ring open in the presence of human plasma. The five-membered lactams **18**–**21** proved the most potent, possessing IC_50_ values of 1.17–6.87 μM against recombinant perforin. All six-membered lactams (**22**–**25**) had IC_50_ values of >20 μM, while indole and 1-methylindole connected at the 5-position (not shown) had moderate activity: IC_50_ = 5.80 and 7.58 μM, respectively. With the *N*-methylindolinone-containing compound **18** established as the best compound (IC_50_ = 1.17 μM), four thiophene replacements (2,5-furan, 2,4-thiazole, 2,5-thiazole, and 2,5-pyridine) were surveyed on this template; however, all were at least 10-fold less potent than **18**.

Four compounds (**18**–**21**) were selected to test for their inhibition of NK cell activity. All showed >80% inhibition of perforin delivered by NK cells with virtually no toxicity (viability 93–96%), with the lead compound **18** delivering 88% inhibition at 93% viability. The same four compounds were also subjected to preliminary physicochemical characterization to determine their potential for *in vivo* pharmacokinetic studies. The solubilities of the sodium salts in water at room temperature were found to be a considerable improvement on the diarylthiophenes with results ranging between indolinone **21** at 392 μg/mL and isoindolinone **19** at 12 940 μg/mL. Stability was tested in water (20 °C, 24 h; 81–97% parent remaining) and in the presence of mouse (42–92%), rat (79–99%), and human (58–85%) microsomes for 30 min. All compounds also had very high levels of mouse plasma protein binding, ranging between 99.70 and 99.97%, likely due to the mildly acidic nature of the benzenesulfonamide NH. Finally, the lead compound **18** was tested for activity against PI3Kα, giving an IC_50_ value of >6.25 μM and confirming that this off-target activity had not been introduced along with the 2,4-benzenesulfonamide side chain. This study successfully established a rationale for the benzenesulfonamides as potent, soluble, and nontoxic candidates for *in vivo* pharmacokinetic studies, with the ultimate aim being progression into a murine model where selective inhibition of perforin could block rejection of transplanted allografts.

**Figure 9:**
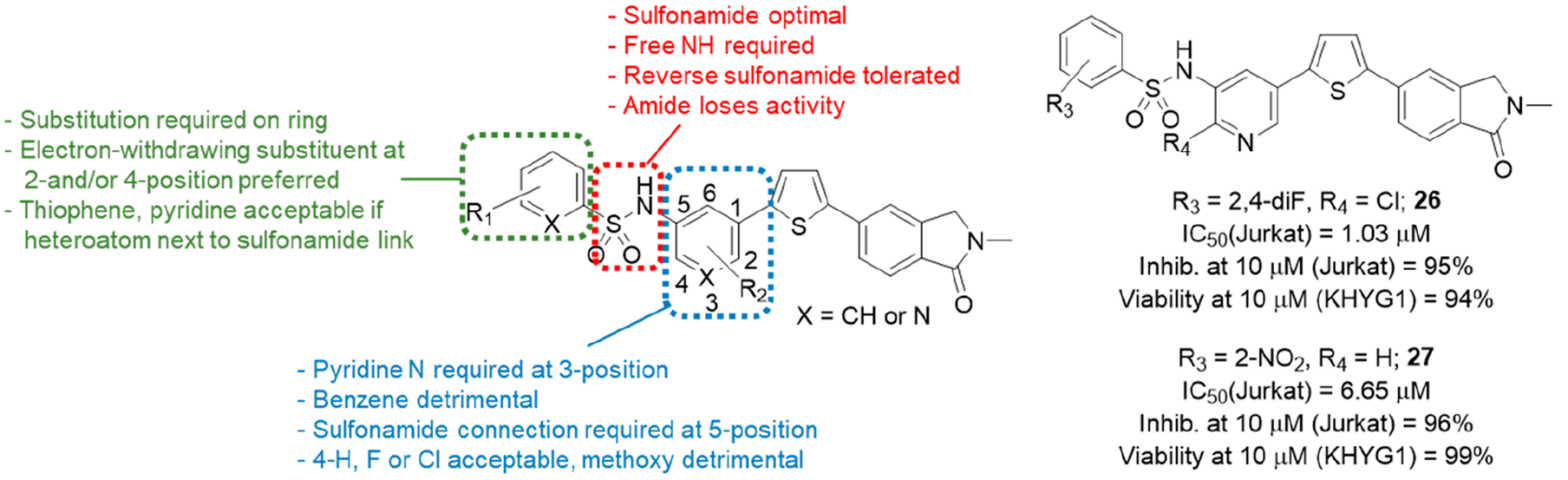
Summary of SAR for benzenesulfonamide and pyridine and preferred analogues **26** and **27**.

The next publication in the series investigated whether it was possible to modulate the activity and physicochemical properties of this template through variation of the sulfonamide linker, linker position, and substitution on the central pyridine and the terminal benzene (Figure 9).^82^ The study began with the sulfonamide linker (red, Figure 9), where it was found that the 5-position was preferred over the 4-position for attachment to the pyridine, while the 3-position was optimal for the pyridine nitrogen. With the appropriate vector for the sulfonamide established, several variations were then explored. The NMe sulfonamide was prepared, resulting in loss of activity and indicating the need for a free NH. The “reverse” sulfonamide was found to be acceptable (IC_50_ = 1.17 μM for lead **18** compared to IC_50_ = 2.24 μM for the reverse isomer); however, replacement of the sulfonamide with an amide resulted in an inactive compound.

With the sulfonamide linker and position of connection optimized, attention turned to a small number of substituents on the central pyridine ring (blue, Figure 9). Replacement of the entire pyridine with benzene was detrimental (IC_50_ = 5.74 μM), but the 4-fluoro- and 4-chloropyridine (**26**) analogues had potencies similar to that of the lead **18** (IC_50_ = 1.03 and 1.99 μM, respectively), while 4-methoxy resulted in a slight loss of activity (IC_50_ = 3.56 μM). A small number of substituted benzenes were surveyed; however, these also had poor activity. The study then progressed to the benzene ring on the left-hand side of the sulfonamide which until this point was comprised of either 2,4-difluorobenzene or 2-pyridyl. With the rest of the molecule fixed, alternative substituents on this ring were explored for effects on potency and physicochemical properties (green, Figure 9). Fifty-two new substituted benzene or aryl sulfonamides were prepared. Substitution on the benzene ring was found to be required, with the parent unsubstituted compound suffering a 7-fold loss of activity compared to **18**. Halogen (F, Cl, Br), methoxy, trifluoromethoxy, trifluoromethyl, cyano, carboxyl, nitro, and methylsulfone (and some selected combinations) were all explored at the 2-, 3-, and 4-positions. Interestingly, when 2,4-difluoro **18** was separated into its constituent 2- and 4-monofluoro derivatives, it was revealed that the contribution of the 2-position was most important (IC_50_ = 2.03 μM), followed by the 4-position (9.65 μM). The 3-fluoro analogue was found to lose activity entirely with an IC_50_ value of >20 μM. This pattern was broadly replicated through all the monosubstituted examples, with electron-withdrawing groups in the 2- and/or 4-position providing the best activity against isolated recombinant perforin. The benzene was also replaced with a small number of heterocycles, of which the 2-pyridyl and 2-thiophene were most potent (both IC_50_ = 1.07 μM) and the corresponding 3-pyridyl and 3-thiophene were relatively poor (IC_50_ = 15.13 and 12.51 μM, respectively), demonstrating a preference for the heteroatom to be located next to the sulfonamide bond.

As in the previous studies, selected examples were then tested for their ability to block the NK cell mediated lysis of labeled target cells, with the concentration reduced to 10 μM from 20 μM due to the improved potency of this series. All analogues with IC_50_ < 2 μM against recombinant perforin also inhibited NK cell activity by 81–95% at 10 μM with 92–100% NK viability, compared to the previous lead **18** which inhibited the NK cells by 68% at 10 μM (100% viability). The one exception was 2-nitro compound **27** which had a modest IC_50_ value of 6.65 μM but the best inhibition of all the selected analogues—96% (99% NK viability). Solubility (water) and stability (water and mouse, rat, human and microsomes) were determined for nine compounds, of which seven were found to be sufficiently soluble and stable for the determination of *in vivo* PK parameters. Plasma pharmacokinetics were measured in male CD-1 mice after the compounds were dosed at 10 mg/kg in a solution of 20% hydroxypropyl-β-cyclodextrin (HPCD) by intraperitoneal (IP) injection. A liquid chromatography/tandem mass spectrometry (LC–MS/MS) based method was utilized to quantitate the parent compound concentration over time. On the basis of *in vitro* potency, solubility, stability, and *in vivo* PK characteristics (*T*_1/2_, *C*_max_, AUC), compounds **18**, **26**, and **27** were identified as potential candidates for progression to *in vivo* efficacy testing to determine their ability to prevent transplant rejection in a mouse model.

**Figure 10:**
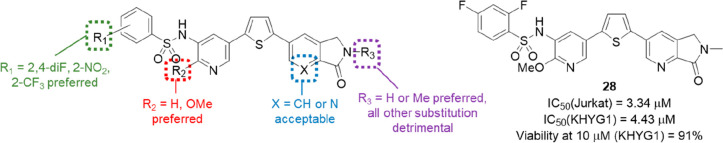
Summary of SAR for aza-isoindolinones and preferred analogue **28**.

The above studies established that the benzenesulfonamide SAR for inhibition of isolated recombinant perforin was very tight, with limited capacity for significant structural change. This led to a focus on more subtle changes designed to optimize physicochemical and pharmacokinetic properties. A small set of 15 novel compounds designed to reduce cLogP and improve solubility was designed and synthesized (Figure 10).^83^

Initial efforts involved introduction of a “solubilizing” side chain appended to the isoindolinone nitrogen (R_3_, purple, Figure 10); however, this strategy failed, with loss of activity observed for all examples. Attention then turned to the insertion of a nitrogen in the isoindolinone ring system to give a series of dihydropyrrolo[3,4-*b*]pyridinones (blue, Figure 10). The previously established SAR was reexamined in the context of this aza-indolinone scaffold, and the approach was found to be effective for retaining inhibitory activity against both isolated perforin and NK cells while simultaneously improving physicochemical properties. *N*-Methyl-aza-indolinones (X = N, R_3_ = Me) were found to be preferred, while the most potent compounds on this template were the 2-CF_3_ (R_1_) derivatives, where R_2_ = H or methoxy, with IC_50_ = 1.38 and 0.78 μM, respectively, against isolated perforin and IC_50_ = 3.30 and 2.21 μM for KHYG1 NK cells. Other compounds with notable activity included R_1_ = 2-nitro and R_2_ = OMe, with IC_50_(Jurkat) = 2.30 μM and IC_50_(KHYG1) = 1.60 μM; and R_1_ = 2,4-difluoro and R_2_ = OMe, with IC_50_(Jurkat) = 3.34 μM and IC_50_(KHYG1) = 4.43 μM (**28**). All four of these compounds were nontoxic, with 91–100% survival of the NK cells.

The key objectives of reducing cLogP and improving solubility were also achieved. For example, where compound pairs were available for direct comparison, the isoindolinones possessed cLogP values of 2.70–3.48, whereas values for the corresponding dihydropyrrolo[3,4-*b*]pyridinones ranged between 2.32 and 2.99. The solubilities of the dihydropyrrolo[3,4-*b*]pyridinone sodium salts were determined in water and were found to be between 1.13 and 20.3 mg/mL. *N*-Methyl-aza-indolinones were found to be 20–50-fold more soluble than the corresponding *N*-methyl-indolinones; however, the reverse was the case for the NH examples where a 3–9-fold loss of solubility was observed. All eight aza-isoindolinones were tested for stability in water and in the presence of microsomes as described above, resulting in four preferred candidates that were found to have very high plasma protein binding (99.89–99.94%) and were Ames negative. Plasma pharmacokinetics for the four compounds were measured in male C57BL/6 mice (to match the strain for the proposed efficacy studies), with dosing at 50–150 mg/kg (0.75 of the previously determined maximum tolerated dose) in a solution of 20% hydroxypropyl-β-cyclodextrin (HPCD) by ip injection. Compound **28** was found to have the longest half-life (18.9 h), an acceptable *C*_max_, and the greatest AUC of the set. Given the extremely high levels of plasma protein binding, the *in vitro* and *in vivo* unbound plasma concentrations of **28** were also compared to confirm that free drug concentrations were sufficient to achieve a therapeutic effect. At a dose of 90 mg/kg it was found that the unbound plasma concentrations of **28** exceeded the unbound *in vitro* IC_50_ for approximately 5–6 h after dosing, sufficient to proceed to efficacy testing. By taking together all the data collected for the benzenesulfonamide series, compounds **27** and **28** were selected as the candidates with the best balance of properties appropriate for testing in *in vivo* efficacy models. These studies are described in section 6.1.1.

## 5 Prodrugs of Perforin Inhibitors

One of the ultimate challenges in drug development is how to achieve optimal drug delivery to the intended site of action without compromising potency toward the target protein, in this case perforin. Due to their rigid and planar structures, many of the published small molecule perforin inhibitors are poorly soluble in water.^67,68,78^ A prodrug approach is a highly effective technique for temporarily changing the physicochemical character of a small molecule to optimize the pharmacokinetic profile and facilitate targeted drug delivery.^84−87^ For delivery of the active small molecule at the target site, prodrugs are required to be bioconverted via either an enzymatic or chemical reaction. However, this may also occur prematurely, i.e., during first-pass metabolism, which may limit the successful application of prodrugs if site-selective release is not optimized early in the development phase. Moreover, species differences in enzyme activity and localization between humans and rodents have made the translation of preclinically developed prodrugs to successful clinical application very challenging.^70^ Nevertheless, there are already many prodrugs for a variety of different therapeutic indications on the market today, and it has been estimated that approximately 10% of all drugs approved worldwide are prodrugs. Furthermore, 11% (33/287) of new small molecular entities approved by the FDA in 2008–2018 were prodrugs.^84^

**Figure 11:**
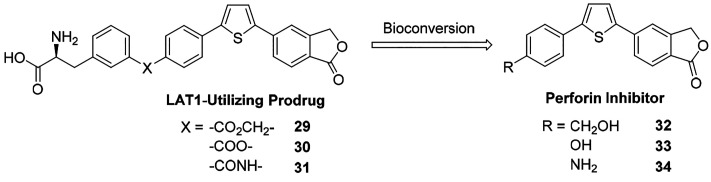
Examples of parent and prodrug perforin inhibitors.

**Figure 12:**
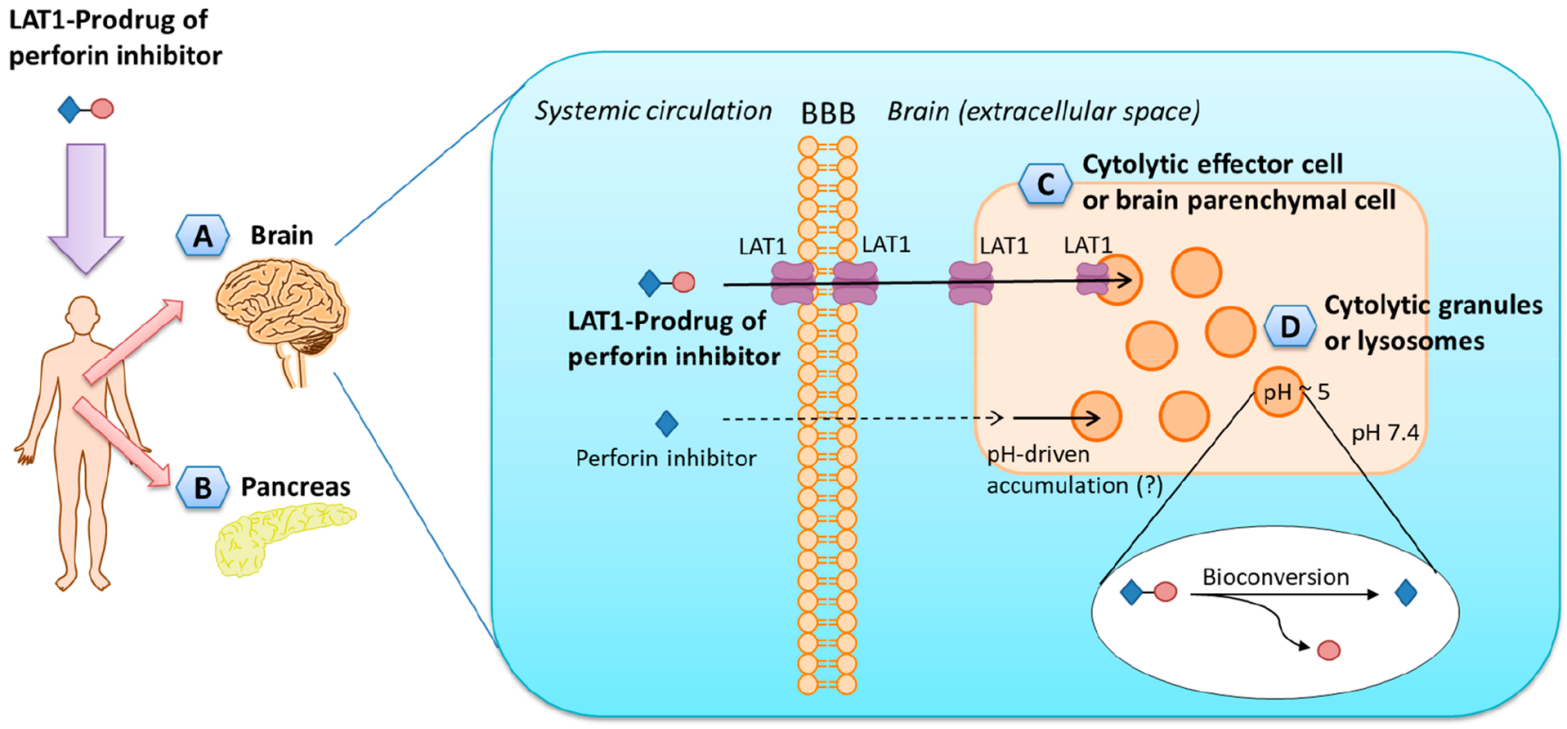
Schematic illustration of pharmacokinetics and targeting properties of the LAT1-utilizing prodrug of perforin inhibitor. The prodrugs can be accumulated via LAT1 into the (A) brain and (B) pancreas. In the brain, the LAT1-utilizing prodrugs can further accumulate into the (C) cytolytic effector cells, or brain parenchymal cells (expressing LAT1), and intracellularly into the (D) cytolytic granules or lysosomes, in which the prodrug is bioconverted to release the active perforin inhibitor. The perforin inhibitor itself cannot be delivered into the brain, although it can be accumulated into slightly acidic organelles most likely due to the pH difference.

Since abnormal perforin activity has been implicated in a wide variety of human diseases, numerous drug delivery methods could potentially be employed, depending on the nature of the therapeutic indication. One approach to achieve site-selective and/or improved drug delivery is the use of prodrugs targeted to membrane transporters. l-Type amino acid transporter 1 (LAT1) is highly expressed at the blood–brain barrier (BBB)^88−90^ and has been utilized to improve delivery of perforin inhibitors into the brain for the treatment of neuroinflammation associated with neurodegenerative diseases and also acute conditions such as stroke and viral brain infection.^91,92^ Reactive astrocytes derived from in and around areas of inflammation in post-mortem brains of patients with multiple sclerosis (MS), Alzheimer’s disease (AD), and Huntington’s disease (HD) have been found to contain perforin throughout the cytoplasm (as opposed to granules as in CTLs and NK cells). Perforin in this context has been postulated to contribute to neuroinflammation, although its exact role in the pathology of these brain diseases is not yet fully understood.^93^ LAT1-utilizing prodrugs of perforin inhibitors (Figure 11) have improved permeation across the BBB and consequently increased brain exposure as demonstrated in pharmacokinetic studies (in contrast, the parent perforin inhibitors were not detected at all in the brain). In addition, prodrug **29** was found to accumulate in the parenchymal cells—cortical neurons, astrocytes, and microglia (Figure 12A,B)—an expected finding since LAT1 is also highly expressed on the plasma membrane of these cell types.^94^

This increased cellular bioavailability was also demonstrated to improve the pharmacological effects of perforin inhibitor **32**, as evidenced by reduced production of caspase-3/-7 (63–68%), lipid peroxidation products (31–46%), and prostaglandin E_2_ (PGE_2_; 55–68%) in a lipopolysaccharide (LPS)-induced neuroinflammation mouse model (250 μg/kg × 3 days).^95^ The prodrug and/or its released parent drug was also found to inhibit the activity of the β-site amyloid precursor protein (APP) cleaving enzyme 1 (BACE1; 72–82% at 1 μM concentration) and acetylcholinesterase (AChE; IC_50_ = 8–12 μM) *in vitro*; therefore, it could have applications in combating neuroinflammation, oxidative stress, and neural apoptosis within the brain for diseases such as AD. However, further investigations are required to determine the exact mechanisms of how perforin inhibitor prodrugs elicit their multifunctional effects.

The above-mentioned LAT1-utilizing perforin inhibitor prodrugs are amino acid mimicking compounds that are 4–15 times more water soluble (1.70–8.14 mg/mL) than their parent perforin inhibitors (0.17–1.98 mg/mL).^91^ They were demonstrated to accumulate into rat liver lysosomes which are slightly acidic, akin to the secretory vesicles of NK cells and CTLs,^96,97^ suggesting that LAT1-utilizing prodrugs of perforin inhibitors could be delivered intracellularly into the granules where perforin is stored (Figure 12C). However, the expression of LAT1 in the secretory vesicles of cytotoxic cells would first need be evaluated, since it is known that lysosomes express LAT1,^98^ which is likely the main reason for high lysosomal accumulation of the LAT1-utilizing prodrugs of perforin inhibitors. However, it is also probable that pH contributes to lysosomal accumulation since the parent perforin inhibitors were also found to be trapped by lysosomes. Regardless, the LAT1-utilizing prodrugs efficiently released their parent perforin inhibitors in lysosomes, either enzymatically or due to the acidic environment.

Upon activation, CD8^+^ T cells have been shown to upregulate their LAT1 expression^99^ although it is not known whether LAT1 in these cells is located only on the plasma membrane or also in intracellular membranes such as secretory granules. This suggests that perforin inhibitors may not need to be delivered to specific tissues if they are able to accumulate in CTLs already in systemic circulation. CTLs could then transport the compounds to the site of action, as cell-mediated carriers.^100^ On the other hand, reaching the cytolytic cells and/or secretory vesicles is not a definitive requirement for the successful delivery of perforin inhibitors. If they are present at the target site, they could also inhibit perforin oligomerization and pore formation extracellularly at the synaptic cleft. Moreover, if inhibitor, perforin, and granzymes are internalized together via endocytosis, this could also prevent apoptosis of the target cell. Thus, the ability of perforin inhibitor prodrugs to access key tissue types through multiple routes could significantly improve the efficacy of this approach.

In the most recent pharmacokinetic study, the prodrug of a perforin inhibitor was highly accumulated in the pancreas,^101^ likely due to the relatively high level of LAT1 expression in this organ (Figure 12D).^102^ Prodrug treatment (30 μmol/kg) was found to decrease pancreatic caspase-3/-7 activity (52%) in mice with LPS-induced pancreatitis (250 μg/kg × 3 days). It is known that, once released from CD8^+^ T cells, perforin is responsible for the autoimmune destruction of pancreatic β-cells in type 1 diabetes mellitus (T1DM).^41,103,104^ Since this prodrug has the ability to infiltrate both the brain (as discussed above) and pancreas at the same time, it has the potential to be evaluated as a dual acting drug candidate in diseases such as T1DM where there are neurodegenerative comorbidities. One potential challenge of transporter-mediated drug delivery is that transporters can have overlapping substrate specificities, which may impair the targeting effect to the selected tissues or cell types. However, since LAT1 is a high affinity–low capacity transporter, if the prodrug transport process is slow, other low affinity–high capacity transporters such as organic anion transporting polypeptides (OATPs) can also participate in prodrug delivery.^91,105^ Depending on the transporter subtype (e.g., OATP1A2 and 2B1, which are highly expressed in the brain), this may even increase the overall brain uptake.^106^

Taken together, the studies above provide proof of concept that the delivery of perforin inhibitors to the tissue/site of action via a prodrug approach is feasible and may have beneficial effects on the pharmacological response at many different levels.

## 6 Activity of Perforin Inhibitors *In Vivo*

### 6.1 Preservation of Bone Marrow Stem Cell Transplants

Perforin inhibitors have been investigated in the context of hematopoietic stem cell transplantation, which is used to treat hematological cancers and nonmalignant disorders such as bone marrow failure and inherited immunodeficiency disorders.^83,107,108^ In the early stages, rejection of immunologically mismatched grafts is known to be driven by the recipient’s NK cells, which predominantly use the perforin/granule exocytosis pathway to kill the transplanted bone marrow stem cells. NK cells are capable of eliminating over 85% of donor leukocyte antigen (HLA) mismatched stem cells within 48 h.^109−111^ While these patients receive pretransplant radio- and chemotherapeutic conditioning to eliminate recipient immune cells, because NK cells are radiation resistant^112^ and immunosuppressant resistant,^109^ a residual population can still contribute to delayed engraftment and graft failure.^113^ Inhibition of perforin activity in the 4–5 days immediately after transplantation could potentially improve engraftment, reduce susceptibility to infection post-transplant, and minimize the use of less selective immunosuppressive agents.

#### 6.1.1 Efficacy and PK/PD Relationships of Benzenesulfonamide Perforin Inhibitors

A set of eight benzenesulfonamides (described in section 4.2.5) were selected as candidates in a preclinical mouse model to test for the preservation of transplanted MHC-mismatched bone marrow cells.^80,82,83^ The compounds were chosen on the basis of their ability to block perforin-mediated lysis *in vitro* and possessed IC_50_ values of 1.03–6.65 μM against recombinant perforin (Jurkat assay) and IC_90_ values of 1.86–30.9 μM in whole NK cells (KHYG1 assay). Candidates were then subjected to pharmacokinetic characterization and tested for *in vivo* efficacy. This resulted in the identification of the most suitable exemplar for which a pharmacokinetic/pharmacodynamic (PK/PD) relationship was developed to inform future *in vivo* efficacy studies.^108^

In the first instance new and published plasma protein binding and mouse pharmacokinetic data was assembled for all eight compounds to enable back-to-back comparisons. All examples were found to be >99.5% bound to mouse plasma proteins. Pharmacokinetic parameters were in the ranges *C*_max_ = 9.8–236 μM, AUC = 220–2885 μM·h, and *T*_1/2_ = 2.5–12 h, and a maximum (single) tolerated dose (MTD) was determined for each compound. With this data in hand, all compounds were progressed to a short-term *in vivo* killing assay that served as a model for CTL/NK-mediated graft rejection during allogeneic bone marrow stem cell (BMSC) transplantation and enabled rapid testing of perforin inhibition *in vivo*. Briefly, equal numbers of allogeneic immunologically mismatched and syngeneic immunologically matched (internal control) bone marrow stem cells were transplanted into mice. The inhibitors were administered intraperitoneally at 0, 24, and 48 h prior to and 18 h after cell transfer with a dose at or near the predetermined MTD. With C57BL/6 perforin-deficient mice (100% perforin inhibition) and untreated C57BL/6 mice (0% perforin inhibition) used as controls, survival of the allogeneic cell fraction was determined in both spleen and peripheral blood 24 h after transplant.

Three of eight compounds produced significant increases in allogeneic bone marrow survival in the spleen, between 48 and 80% of that observed in the perforin-deficient control mice. The rank order of perforin inhibition *in vivo* did not follow the exact order observed in the *in vitro* KHYG1 assay; however, those compounds with IC_90_ values of <5 μM were confirmed as effective inhibitors of perforin *in vivo*, supporting the use of this *in vitro* assay as a screen for *in vivo* candidates. Some analogues were also associated with a reduction in splenocyte cellularity; thus, the two most potent inhibitors that did not produce this effect (**18** and **27**) were selected for a dose–response study at 100, 75 and 25% of MTD. Compound **27** was ultimately chosen for the evaluation of a PK/PD relationship because it produced a more linear dose-dependent increase in allogeneic bone marrow survival than **18**. Given the suggestion of splenic toxicity in some analogues at a single dose, for the avoidance of doubt **27** was investigated for its impact on the spleens of unmanipulated naive mice at 100 and 150 mg/kg by use of an extended dosing strategy. No weight loss, change in spleen or blood cell counts, or reduction in splenic CD4^+^ or CD8^+^ T cells and CD19^+^ B cells or NK1.1^+^ NK cells was observed compared to the vehicle control, suggesting any splenic atrophy was most likely due to the bone marrow transplantation procedure.

**Figure 13:**
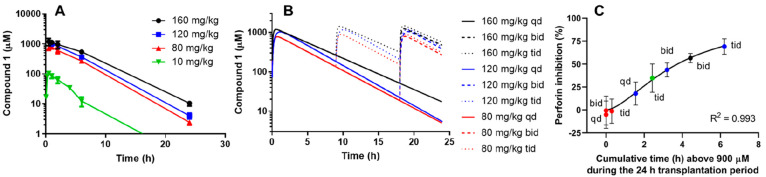
Compound **27** mouse pharmacokinetics and efficacy. (A) Plasma concentration–time profiles of multiple dose levels of **27** by single dose. (B) Simulated plasma concentration–time profiles of different multiple dose schedules of **27** based on a one-compartment pharmacokinetic model. (C) Pharmacokinetic/pharmacodynamic relationship for compound **27** that showed the strongest PK/PD correlation was percent perforin inhibition *in vivo* (peripheral blood) and the time that total plasma concentrations remained above 900 μM. Modified from ref (108). Copyright 2022 American Chemical Society.

Compound **27** mouse pharmacokinetic parameters were then determined from treatment of mice at 10, 80, 120, and 160 mg/kg, with blood samples collected at multiple time points after dosing. Overall, a linear dose-dependent relationship, was found and while initially millimolar concentrations of drug in the plasma were reached, these levels were negligible by 24 h, giving an elimination half-life of 2.4–3.4 h across the different dose levels (Figure 13A). This data was then used to simulate different dosing strategies to predict optimal dose levels and schedules for the pharmacodynamic study (Figure 13B). The accuracy of this model was then confirmed by testing at specified doses and measuring the resulting plasma drug concentration at a range of time points over 24 h. This data showed a close correlation with the simulated data, indicating that the plasma pharmacokinetics of **27** can be fitted by using a one-compartment model.

Following on from the single dose studies, multiple schedules and doses of compound **27** were then tested in the *in vivo* bone marrow transfer assay to examine whether more frequent dosing could improve efficacy. The percentage of perforin inhibition compared to the perforin-deficient control mice was calculated for both peripheral blood and spleen, and it was correlated with plasma *C*_max_, AUC, *C*_min_, and a high arbitrarily chosen concentration of 900 μM in order to find the PK parameter that best predicted *in vivo* efficacy. For both peripheral blood and spleen the strongest correlation was observed between percent perforin inhibition *in vivo* and the time that total plasma concentrations exceeded 900 μM (Figure 13C). It was concluded from this PK/PD relationship that achieving an initial high *C*_max_ was not sufficient for optimal perforin inhibition in the *in vivo* mouse model and that elevated plasma drug concentrations of **27** (>900 μM) needed to be maintained continuously. Due to the high levels of plasma protein binding (99.8%) for compound **27**, it was postulated that saturation of binding may occur and that this would help explain why time above 900 μM was most strongly associated with efficacy. The binding level of **27** to proteins in the medium used for the KHYG1 assay was determined and used to estimate an unbound IC_90_ value of 0.77 μM. On the basis of this figure, together with plasma protein binding of 99.8% and the requirement for an *in vivo* plasma concentration of 900 μM for efficacy, it was calculated that the concentration required for efficacy should be maintained at 3 times the unbound IC_90_ for as long as possible within the transplant window. Given the short elimination half-life of these benzenesulfonamide compounds, it was concluded that either frequent administration at high doses or intravenous administration would be required to maintain sufficiently high concentrations to induce perforin inhibition for as long as possible.

Another approach to enable maintenance of the concentration above 3 times the unbound IC_90_ with only a single daily dose for the full 24 h transplantation window was to identify a benzenesulfonamide with a longer elimination half-life. Compound **28**, discussed in section 4.2.5, achieved this criterion with a *T*_1/2_ of 18.9 h.^83^ The *C*_max_ and AUC also exceeded those of the eight compounds^108^ tested previously (1310 μM and 42 475 μM·h, respectively). When **28** was tested for efficacy at 90 mg/kg in a 24 h assay to determine perforin-dependent NK cell mediated graft rejection, a 66% reduction of perforin activity was observed, resulting in preservation of the transplanted mismatched target cells. The compound was well tolerated, was adequately soluble, and was found to largely overcome the problems that had afflicted the earlier analogues.

### 6.2 Fulminant Viral Hepatitis

As discussed above, a fatal CTL-mediated immunopathology that might be responsive to perforin inhibition is the “posthepatitic” liver failure that idiosyncratically affects a small minority of patients who contract serious viral hepatitis, most commonly that caused by hepatitis B. While most patients show only transiently elevated serum levels of liver transaminases and recover within 3–7 days, a few experience uncontrolled loss of hepatocyte mass due to immune attack of the liver, as reflected in extreme and persistently high transaminase levels. This leads to liver failure that is fatal unless managed expertly in an intensive care ward.

It has recently been shown that compound **27**, delivered to mice twice daily at 100 mg/kg over a short window of time (2 days) commencing several days *after* virus infection, was able to prevent the death of virtually all the infected mice, whereas 100% of mice that received only the drug diluent succumbed.^38^ As human hepatitis B virus does not cause serious infection in mice, a pathogenic hepatotropic adenovirus expressing the extrinsic antigen chicken ovalbumin (OVA) was used for these studies. This virus infects only hepatocytes (not other types of liver cell), and cell damage/death causes the release of liver transaminases into the circulation, as in human hepatitis B and adenovirus infection. OVA expression in the infected cells is a useful feature of the model, as this foreign antigen elicits the recruitment of CTLs whose number, anatomical location, activation status, and cytotoxic function for the infected cells can be tracked by a variety of tools readily available in many immunology laboratories.

The mice that survived infection in this model did not relapse when the drug was withdrawn, and their survival was associated with a major (∼60%) reduction in peak circulating aspartate transaminase/alanine transaminase levels, despite therapy having commenced well after infection. The key to mouse survival was 2-fold. First, the drug reduced the tempo of virus infection, slowing spread to uninfected cells to the extent that enough healthy hepatocytes remained unscathed to ensure a level of liver function sufficient for mouse survival. The second important factor related to a facet of infection that had not previously been suspected. Consistent with the reduced transaminase levels, histology of the mouse liver showed that drug treatment reduced the number of hepatocytes dying by apoptosis. A further unsuspected positive consequence of treatment was that far fewer CD8^+^ CTLs were observed infiltrating the liver parenchyma. On further investigation, it was found that the reduced CTL infiltrate was due to preservation of CD31^+^ sinusoidal endothelial cells that line small blood vessels in the liver. Although these cells are resistant to virus infection, it was demonstrated that they very efficiently take up and represent OVA released by neighboring dying hepatocytes. As a result, the endothelial cells, which normally restrict the migration of activated T cells into the liver became “secondary targets” for CTL attack. Death of these protective cells was associated with increased CTL infiltration and more rapid hepatocyte death. Conversely, blocking the death of sinusoidal endothelial cells by perforin inhibition restored blood vessel integrity and attenuated the death of hepatocytes. Histologically, this was demonstrated by orderly CD31 staining restored in the sinusoidal channels in the livers of perforin inhibitor treated mice.^37^

### 6.3 Colitis

The most widely used food colorants in the world, azo dyes Red 40 (Allura Red AC) and Yellow 6, have recently been reported to trigger an inflammatory bowel disease (IBD)-like colitis in mice that possess increased IL-23 signaling. Colitis development was shown to depend on activated CD4^+^ T cells, identifying Red 40 in particular as an environmental risk factor capable of triggering IBD-like colitis in R23FR mice.^114^ Perforin inhibitor **27** was subsequently used to show that the colonic pathology in this mouse model was related to initiation of colonic inflammation by CD4^+^ T cells via a mechanism that did not require active perforin but was instead mediated by FasL promoted caspase-dependent cell death.^115^

## 7 Concluding Remarks and Future Perspectives

Perforin has been shown to be a novel and tractable target, the inhibition of which would address a significant unmet need in NK- and CTL-mediated immune disorders. An immunosuppressant targeting perforin would provide the first-ever therapy focused specifically on one of the principal cell death pathways leading to transplant rejection and a range of autoimmune diseases. Perforin has many of the ideal characteristics of a target for therapeutic intervention. It controls numerous critically important types of immune pathology and yet is encoded by a single copy gene with no redundancy of function. If perforin is absent or blocked *in vivo*, nothing else can substitute for it; the evidence for this fact is powerful in both mice and humans.

Perforin does present challenges as a target for a drug discovery campaign because it is not an enzyme such as a kinase or protease, where blocking a catalytic site is normally the goal, so the classic kinetics of drug:target interactions do not apply. Indeed, perforin’s precise mechanism of action is still not fully understood at the molecular level, and the cell biology of perforin killing is only gradually being deciphered. As an example, even the concentration of perforin delivered at the immune synapse is not yet known. The small molecule inhibitors described in this perspective are blocking a complex biological process, and there is limited *in vitro* modeling data available to inform this. In addition to pore formation with perforin, cell death involves many different molecules (such as the granzymes) and cell biological processes (including repair of the target cell membrane) that occur in an incompletely understood space (the immune synapse) and in a poorly defined compartment of the body (chiefly blood, the lymphoid compartment, and the bone marrow). It has been shown that at least some of the inhibitors discussed above possess the capacity to block perforin activity within the immunological synapse without affecting the stability of synapse formation, leading to the conclusion that the inhibitors act on perforin after its release, rather than within the T cell. This raises the likelihood that these inhibitors act by blocking either the binding of the perforin C2 domain to the lipid membrane of the target cell or oligomerization of the perforin monomers, thereby preventing the first steps of pore formation. This in turn means that the drug mode of action is complex, akin to blocking protein/protein or protein/protein/lipid interactions and may well explain why many of the early perforin inhibitors that were identified had unfavorable physicochemical properties such as high lipophilicity and low solubility.

One issue that is frequently raised is whether blocking perforin in humans could result in unforeseen toxicity related to its potential immunoregulatory functions. In this regard, the observation that pharmacological intervention for just 2 days was sufficient to rescue 100% of mice from CTL-induced fulminant liver failure compared to controls^38^ is a heartening result. Simply temporarily “blunting” the CTL effector response to virus rather than total inhibition served to preserve sufficient hepatocyte mass to avoid fatal acute liver failure. A carefully conceived path to the clinic that commences by treating short-term pathologies such as this, Susac syndrome, or cerebral malaria, or that focuses on topical application of drugs for autoimmune skin diseases should adequately obviate these theoretical risks. While it is prudent to delay addressing human diseases that would in principle require long-term perforin inhibition (at least until human toxicity can be assessed in shorter studies), the results of a recent, large population study showed that the 1/600 Caucasian individuals who inherit hypomorphic mutant A91V-perforin in the homozygous state survive to the age of 75 years in normal numbers, with no lifelong predisposition to systemic inflammatory diseases, virus infection, or cancer.^116^

The latest generation of benzenesulfonamide-based perforin inhibitors establish proof of concept that it is possible to successfully block the activity of the granule exocytosis cell death pathway *in vivo*. These inhibitors are highly promising, and it is likely that further testing in mouse models of various CTL-mediated autoimmune diseases, including type 1 diabetes, Susac syndrome, leishmaniasis, and cerebral malaria, will prove similarly fruitful, providing a pathway to a clinical treatment for perforin-dependent immune pathologies.

## Notes

The authors are open to providing samples of perforin inhibitors for academic and industry-based research on a collaborative basis.

## Abbreviations Used

AD: Alzheimer’s disease
APP: amyloid precursor protein
BACE1: β-site APP cleaving enzyme 1
BMSC: bone marrow stem cell
CSV: cytotoxic secretory vesicle
CTL: cytotoxic T lymphocyte
HD: Huntington’s disease
HLA: human leukocyte antigen
HPCD: hydroxypropyl-β-cyclodextrin
IBD: inflammatory bowel disease
LAT1: l-type amino acid transporter 1
MS: multiple sclerosis
mTOR: mammalian target of rapamycin
NK: natural killer
NOD: nonobese diabetic
OATP: organic anion transporting polypeptide
OVA: ovalbumin
PAIN: pan-assay interference
PGE_2_: prostaglandin E_2_
PI: propidium iodide
PI3Kα: phosphoinositide 3-kinase α
PLO: pneumolysin
RBL: rat basophilic leukemia
SAR: structure–activity relationship
SRBC: sheep red blood cells
T1DM: type 1 diabetes mellitus
TRAIL: tumor necrosis factor related apoptosis-inducing ligand
